# Signs of biological activities of 28,000-year-old mammoth nuclei in mouse oocytes visualized by live-cell imaging

**DOI:** 10.1038/s41598-019-40546-1

**Published:** 2019-03-11

**Authors:** Kazuo Yamagata, Kouhei Nagai, Hiroshi Miyamoto, Masayuki Anzai, Hiromi Kato, Kei Miyamoto, Satoshi Kurosaka, Rika Azuma, Igor I. Kolodeznikov, Albert V. Protopopov, Valerii V. Plotnikov, Hisato Kobayashi, Ryouka Kawahara-Miki, Tomohiro Kono, Masao Uchida, Yasuyuki Shibata, Tetsuya Handa, Hiroshi Kimura, Yoshihiko Hosoi, Tasuku Mitani, Kazuya Matsumoto, Akira Iritani

**Affiliations:** 1 Graduate School of Biology-Oriented Science and Technology, Kindai University, Wakayama, 649-6493 Japan; 2 Institute of Advanced Technology, Kindai University, Wakayama, 642-0017 Japan; 3 Department of Mammoth Faunal Studies, Sakha (Yakutia) Republic Academy of the Sciences, Yakutsk, 677077 Russia; 4 NODAI Genome Research Center, Tokyo University of Agriculture, Tokyo, 156-8502 Japan; 5 Department of Bioscience, Tokyo University of Agriculture, Tokyo, 156-8502 Japan; 6 National Institute for Environmental Studies, Ibaraki, 305-8506 Japan; 7 Institute of Innovative Research, Tokyo Institute of Technology, Kanagawa, 226-8503 Japan

## Abstract

The 28,000-year-old remains of a woolly mammoth, named ‘Yuka’, were found in Siberian permafrost. Here we recovered the less-damaged nucleus-like structures from the remains and visualised their dynamics in living mouse oocytes after nuclear transfer. Proteomic analyses demonstrated the presence of nuclear components in the remains. Nucleus-like structures found in the tissue homogenate were histone- and lamin-positive by immunostaining. In the reconstructed oocytes, the mammoth nuclei showed the spindle assembly, histone incorporation and partial nuclear formation; however, the full activation of nuclei for cleavage was not confirmed. DNA damage levels, which varied among the nuclei, were comparable to those of frozen-thawed mouse sperm and were reduced in some reconstructed oocytes. Our work provides a platform to evaluate the biological activities of nuclei in extinct animal species.

## Introduction

Ancient species carry invaluable information about the genetic basis of adaptive evolution and factors related to extinction. Fundamental studies on woolly mammoth (*Mammuthus primigenius*) genes, including whole genome analyses^1–3^, led to the reconstitution of mammoth haemoglobin with cold tolerance^4^ and to an understanding of the expression of mammoth-specific coat colour^5^ and temperature-sensitive channels^6^. Moreover, proteomic analyses have shown the presence of proteins in the remains^7,8^. Meanwhile, the investigation of biological activities of nuclei isolated from the remains using means of nuclear transfer (NT) approach is still in progress.

**Figure 1:**
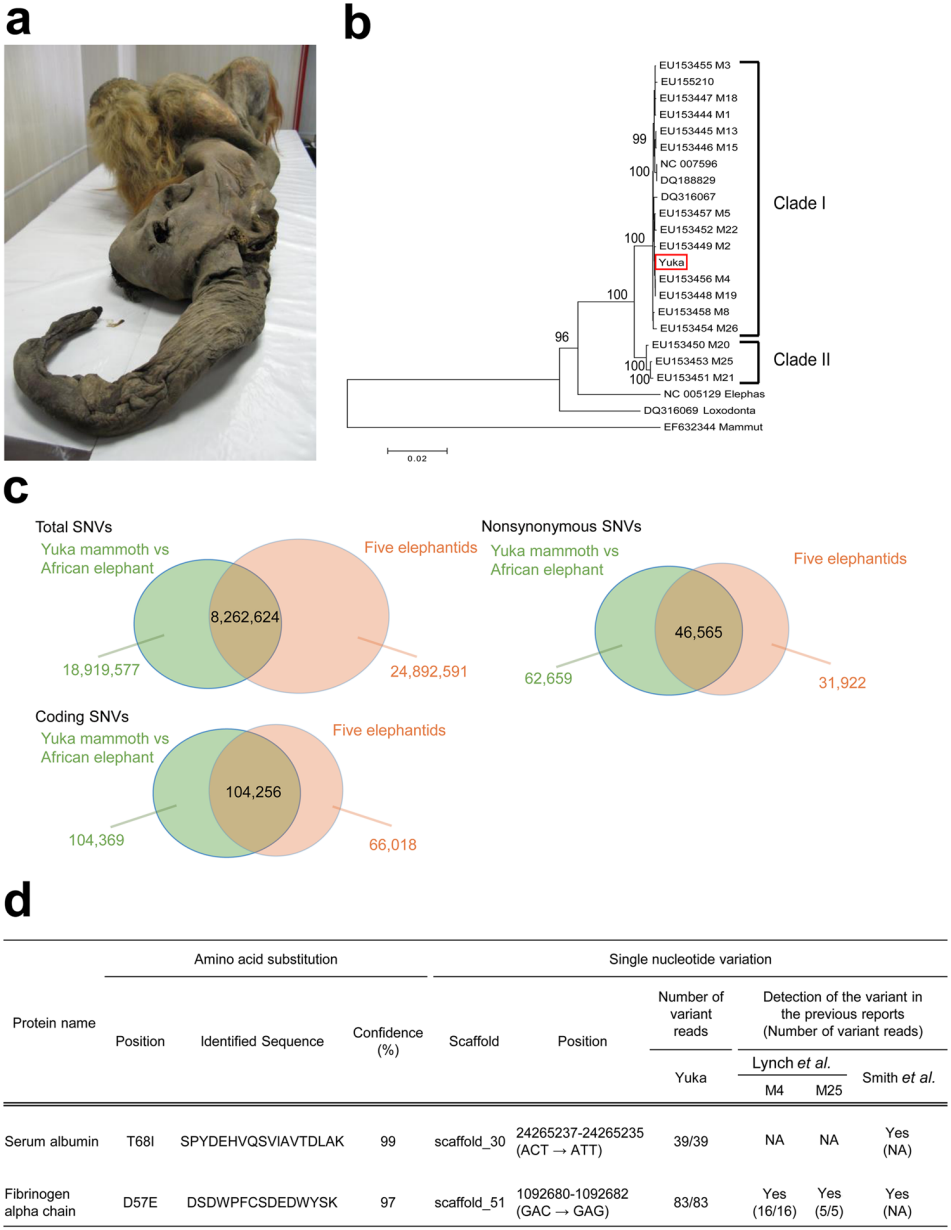
Validation of mammoth sample by whole-genome sequencing. (**a**) Photograph of the remains of a woolly mammoth, named Yuka. (**b**) The tree was inferred using the Maximum Likelihood method based on the Tamura-Nei model in MEGA6. The number at the nodes indicates the confidence level greater than 95% with 1,000 bootstrap replicates. The scale bar represents a distance of 0.02 substitution per site. (**c**) Venn diagrams showing comparison of sequence nucleotide variants (SNVs) between the Yuka mammoth and other elephantid specimens. SNVs identified in at least one of the five elephantid specimens (M4 and M25 woolly mammoths and three Asian elephants) are indicated as five elephantids. (**d**) Amino acid substitution in mammoth serum albumin and fibrinogen alpha chain are compatible with mammoth SNVs found in our genomic sequencing and previous reports^6,15^.

Our initial attempt of NT using 15,000-year-old mammoth tissues resulted in no nuclear reorganisation in mouse oocytes^9^, possibly owing to the technological limitations at that time and the inappropriate state of the frozen mammoth tissues. In the present study, the combination of NT and less-invasive live-cell imaging, previously developed by us^10^, and excavation of other remains of the woolly mammoth from the Siberian permafrost, named ‘Yuka’ (Fig. 1a)^11^ led us to study the biological activities of mammoth nuclei.

Radiocarbon dating suggested that Yuka mammoth was 28,140 (±230) years old. The authenticity of our tissue samples after such a long frozen period was confirmed by whole-genome sequencing. Genomic DNA libraries of the Yuka mammoth remains were constructed using Phusion polymerase, which provides efficient amplification with high fidelity, excluding post-mortem damage (C-to-T or G-to-A substitutions) (Supplementary Fig. S1). We aligned 1,446,220,624 sequencing reads to the African elephant genome (Loxafr3.0) and successfully mapped 747,034,525 reads (Supplementary Table S1). The average mapping rate of reads was 51.7%, and 83.0% of the elephant reference genome was covered by more than one sequence read, yielding 23-fold coverage of the reference genome. Phylogenetic analysis indicates that the Yuka mammoth mitochondrial genome fell into the clade I (Fig. 1b), which contains mammoth specimens found in the large area across the Holarctic^12–14^. Next, we focused on single sequence nucleotide variants (SNVs) by comparing them with those of the five elephantids (M4 and M25 woolly mammoths and three Asian elephants)^6^. Variant calling using SAMtools resulted in 27,182,201 SNVs in the Yuka mammoth genome against the African elephant reference genome. These SNVs showed a significant overlap with those of the five elephantid specimens (Total SNVs: 30%, Coding SNVs: 50%, Nonsynonymous SNVs: 43%) (Fig. 1c)^6^.

**Figure 2:**
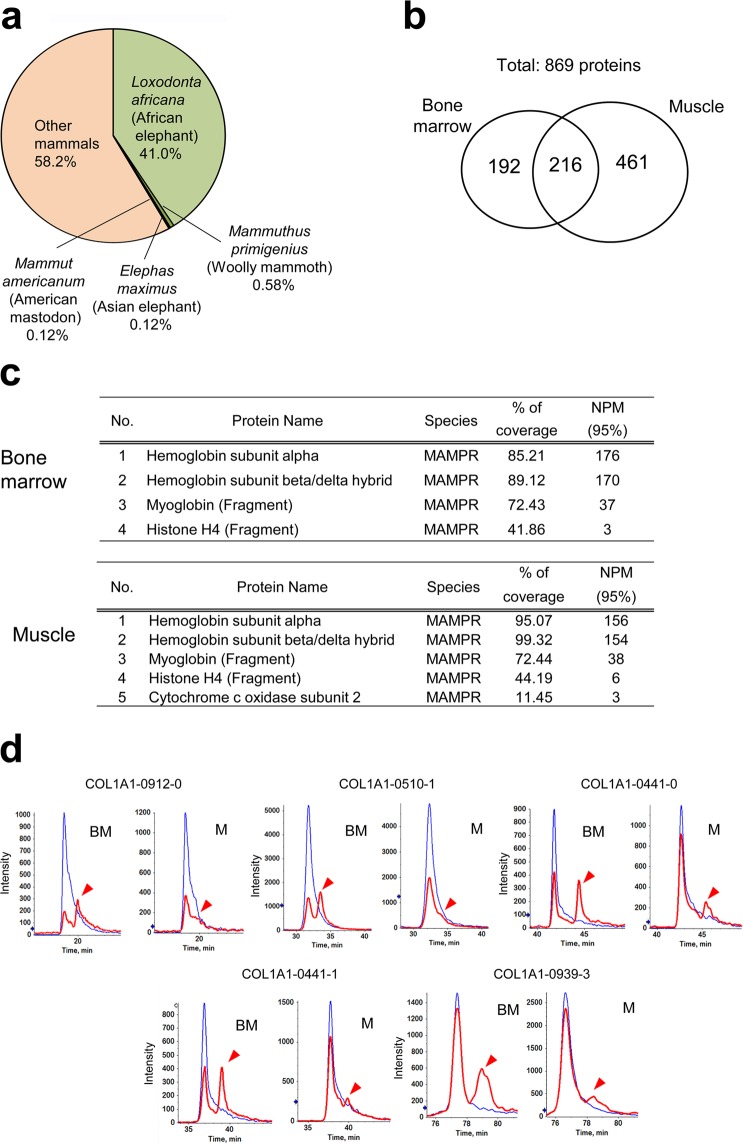
Proteomic analyses of mammoth tissues. (**a**) More than 40% of the proteins identified by searching against mammalian and mammoth protein databases belong to the order Proboscidea. (**b**) The Venn diagram represents the proteins identified in the bone marrow and the muscle tissues. (**c**) The list of the proteins identified by the search against the mammoth protein database. NPM (95%) means the number of peptides matched with confidence of more than 95%. (**d**) Comparison of the degree of glutamine deamidation in five collagen peptides between the bone marrow (BM) and muscle (M) samples using extracted ion chromatography. Blue and red lines correspond to normal and deamidated forms of the collagen peptides, respectively. Arrowheads indicate peaks of deamidated forms.

We then performed proteomic analyses to gain information about the repertoire and modifications of proteins. The data obtained by LC-MS/MS analyses of proteins extracted from the mammoth samples were searched against UniProt mammalian protein database including all proteins predicted from the *Loxodonta africana* (African elephant) genome and 134 UniProt mammoth sequences. Consequently, we identified 869 distinct proteins. Of them, the greatest number of matches was seen in *L. africana* (41%), followed by *Elephas maximus* (Asian elephant) and *Mammut americanum* (American mastodon), and five mammoth proteins were identified (Fig. 2a and c and Supplementary Table S2). As a result, 41.8% of the proteins were identified in the order Proboscidea, into which the woolly mammoth is classified. Among 869 proteins, 408 were identified from the bone marrow sample, whereas 677 were from the muscle sample and only 216 proteins were common to both samples (Fig. 2b), indicating the identification of tissue-specific proteins. The gene ontology analysis identified GO terms related to bone marrow functions such as osteoblast differentiation (*p* = 4.80E-04) in bone marrow samples, and those related to muscle functions such as muscle sliding (*p* = 7.10E-14) in muscle samples (Supplementary Table S3).

To further consolidate evidence of the identification of mammoth proteins, we performed comparative analyses between genomic sequences and our proteomic data. Of the proteins identified via LC-MS/MS as significant identities with the African elephant proteins, we selected one extracellular matrix protein (laminin subunit gamma-1), five serum proteins (fatty acid binding protein 4, serum albumin, serotransferrin, complement factor H and fibrinogen alpha chain) and two intracellular proteins (aminopeptidase and glutathione S-transferase) for further comparative analysis because all of them showed high sequence coverage. All thirteen amino acid substitutions in the eight proteins were found to be compatible with nonsynonymous SNVs described above and reported previously^6,15^ (Fig. 1d and Supplementary Table S4). Therefore, these results demonstrate that our proteomics analyses identified the protein sequences of the Yuka mammoth remains without contaminations from the environment.

Comprehensive analyses of post-translationally modified peptides demonstrated 99 enzymatic- and non-enzymatic modifications, including those not reported previously in ancient animal remains (Supplementary Table S5). Next, we evaluated protein aging of mammoth tissues with the non-enzymatic spontaneous modifications of peptides, because van Doorn *et al*. reported that site-specific deamidation can be used as the marker of protein deterioration^16^. Mammoth samples showed more deamidation of asparagine and glutamine, oxidation of phenylalanine and partial degradation of peptide bonds than elephant samples (*P* < 0.05). These spontaneous modifications were suppressed in the muscle samples compared to the bone marrow samples (Supplementary Table S5). Extracted ion chromatography of the five collagen peptides^16^ showed that the glutamine residues were more deamidated in the bone marrow than in the muscle (Fig. 2d). In addition, we counted all the identified collagen sequences and calculated the degree of deamidation (Supplementary Table S6), which was significantly reduced (*P* < 0.05) in our muscle sample compared to our bone marrow sample and the previously reported Dent and La Sena mammoths but was similar to that of the Siberian mammoth^17^. These data indicate that our mammoth samples, particularly from the muscle, were well-preserved.

Interestingly, the nuclear protein histone H4 was detected, which is reminiscent of the retention of nuclear components in the remains (Fig. 2c). Search against the database of all mammalian species identified other nuclear proteins, such as histones, histone chaperones, proteins implicated in mRNA processing or transport and nuclear membrane proteins (Supplementary Table S2). In addition, we identified two well-characterised epigenetic modifications on histone molecules, methylation of H3K79 and H4K20 (Supplementary Fig. S2A and B), which are involved in transcriptional regulation and genome maintenance^18,19^. Our high-sensitive proteomic analysis suggests that the remains retain nuclear components.

**Figure 3:**
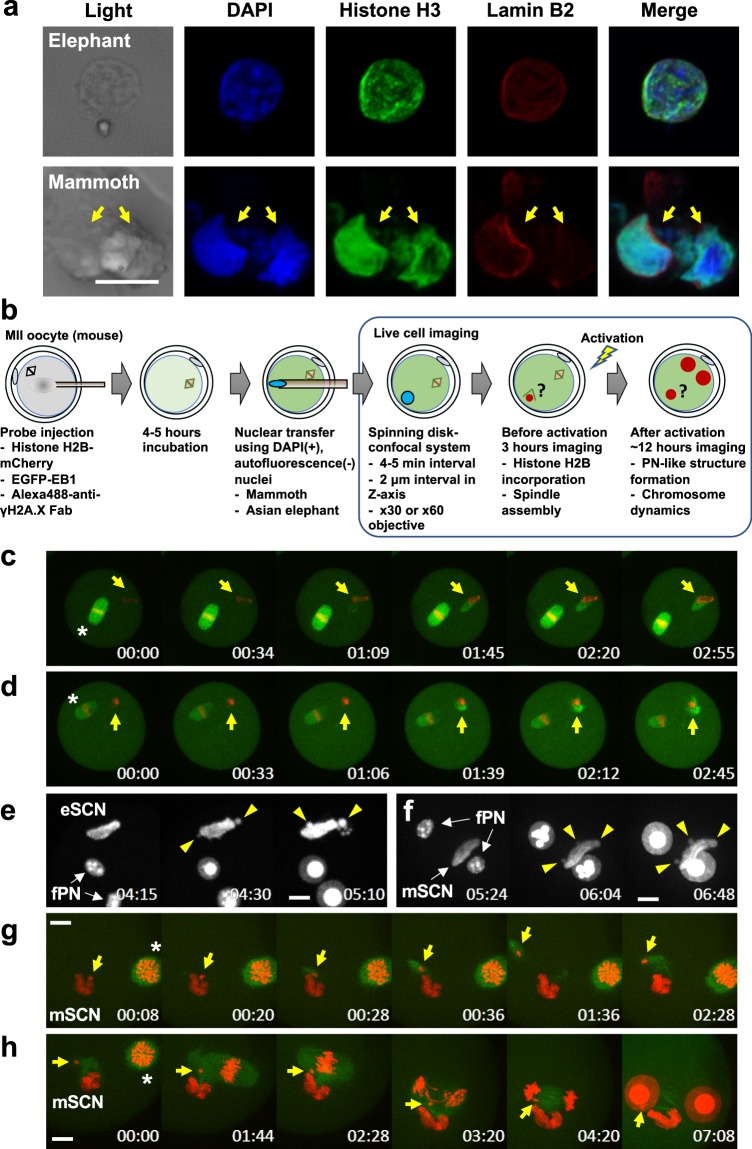
Reconstruction of mammoth nuclei upon nuclear transfer to mouse oocytes. (**a**) Immunostaining of nucleus-like structure from elephant or mammoth tissue with anti-histone H3, anti-lamin B2 and DAPI. (**b**) Experimental scheme and conditions for NT and imaging. Mouse metaphase II oocytes were injected with mixture of various fluorescent probes, subjected to NT without enucleation of metaphase II chromosomes and imaged. (**c**–**h)** Time-lapse images of NT oocytes injected with mammoth (**d**,**f**,**g** and **h**) or elephant (**c** and **e**) nuclei at the stages of before (**c**,**d** and **g**) and after (**e**,**f** and **h**) activation. Green and red colours represent EB1 (spindle) and H2B (chromatin), respectively. White colour represents H2B-mCherry signal (**e** and **f**). Yellow arrows indicate mammoth or elephant nucleus-like structures (**c** and **d**) and the mammoth chromosome that detached from nucleus-like structure and entered into the mouse pronucleus (**g** and **h**). Arrowheads indicate the injected mammoth or elephant nucleus forming pronucleus-like structures (nuclear blebs). Asterisks show metaphase II chromosomes. eSCN, mSCN and fPN represent elephant somatic cell nucleus, mammoth somatic cell nucleus and female pronucleus, respectively. The time (h:min) shown in the images indicates time duration from the starting point of imaging. Bar = 10 μm.

These findings motivated us to seek cell nuclei from the muscle remains. Although DAPI-positive and autofluorescence-negative nucleus-like structures were rarely found (Supplementary Figs S3 and S4), we chose the autofluorescence-negative structures for the subsequent live-cell imaging of nuclear-transferred embryos since autofluorescence disturbs accurate tracing of fluorescent-tagged proteins. In total, 88 nucleus-like structures were collected from 273.5 mg mammoth tissue in 5 independent experiments (Supplementary Table S7). Our immunostaining protocol developed for single suspended cells from remains (Supplementary Fig. S5) revealed that these structures were positive for lamin B2 and histone H3, both of which were identified by mass spectrometry (Fig. 3a and Supplementary Fig. S6), suggesting that cell nuclei are, at least partially, sustained even in over a 28,000 year period.

We asked whether the mammoth nuclei are able to show spindle and nuclear reconstitution upon NT to mouse oocytes by the traceable live-cell imaging technique^10^, using EGFP-EB1^20^ and histone H2B-mCherry as probes (Fig. 3b). To compare the responses of injected nuclei with those of endogenous ones, the metaphase II plate, inherent chromosomes of oocyte, was not removed. Oocyte chromosomes were distinguishable from mammoth nuclei by their morphological characteristics. Namely, typical metaphase II chromosomes of mouse were already observed by EGFP-EB1 and histone H2B-mCherry signals when the imaging had started, while the assembly of the probes onto the injected nucleus took some time (Fig. 3c and d). In addition to mammoth nuclei, cell nuclei from frozen stored post-mortem elephant tissues were injected into mouse oocytes as a control. Soon after NT (Supplementary Fig. S7), H2B-mCherry was assembled onto the injected elephant (Fig. 3c; Supplementary Movie S1A) and mammoth nuclei (Fig. 3d; Supplementary Movie S1B and C). This H2B incorporation was observed in 96% and 88% of NT oocytes injected with elephant and mammoth nuclei, respectively (Supplementary Table S8), suggesting the possibility of the nucleosome formation in most of mammoth nuclei. Furthermore, oocyte-derived spindle was assembled around mammoth nuclei in 21% of the NT oocytes (Fig. 3d; Supplementary Table S8; Supplementary Movie S1C), indicating that some mammoth nuclei can react to mouse kinetochore proteins. No premature chromosome condensation was observed in elephant or mammoth nuclei after NT, probably due to the frozen condition of post-mortem tissues. After activation of those NT oocytes, pronucleus-like structure budded from the injected elephant (yellow arrow heads; Fig. 3e) and even from a mammoth nucleus (yellow arrow heads; Fig. 3f; Supplementary Tables S8 and S9; and Supplementary Movie S2) simultaneously with the timing of mouse pronuclear formation. We define nuclear blebs that were transformed from condensed elephant/mammoth chromatin (Fig. 3e and f, arrows) as pronucleus-like structures since the appearance of the nuclear blebs coincides with female pronuclear formation. Moreover, a part of mammoth chromosomes with spindle was apparently incorporated into mouse pronuclei in NT oocytes (Fig. 3g and h and Supplementary Movies S3 and S4). All reconstructed oocytes were arrested or degenerated during the one-cell stage (all trials are listed in Supplementary Table S9), probably due to the G2/M DNA damage checkpoint. These results indicate that a part of mammoth nuclei possesses the potential for nuclear reconstitution.

**Figure 4:**
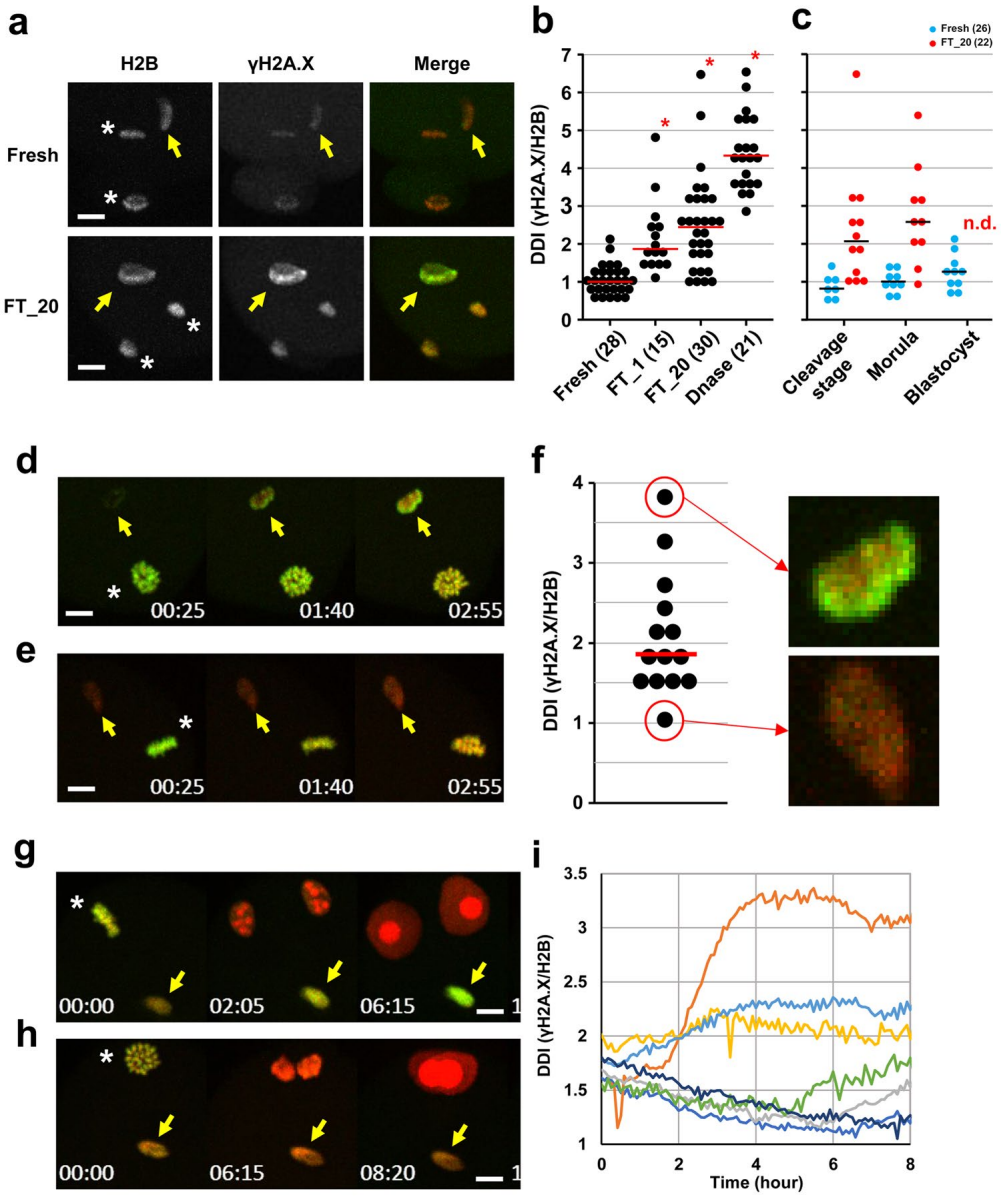
Quantification of DSBs of mammoth genome reveals the various degrees of DNA damage. (**a**–**c**) DSBs in embryos injected with fresh, frozen-thawed once (FT_1) or twenty times (FT_20), DNase-treated sperm. (**a**) Representative images of γH2A.X (green) and H2B (red) signals of fresh and FT_20 sperm injected into mouse oocytes. Arrows and asterisks indicate sperm nuclei injected and anaphase II chromosomes, respectively. (**b**) DDIs (γH2A.X/H2B ratio) of FT and DNase-treated sperm were significantly higher than those of fresh sperm (Steel test, *P* < 0.01). Number of samples analysed are shown in parentheses. (**c**) Developmental abilities of embryos injected with FT_20 sperm were significantly reduced at the blastocyst stage (Fisher exact test, *P* < 0.01) but not at the morula stage (*P* = 0.077). Blastocyst with FT_20 sperm was not detected (n.d.). (**d–i**) DSBs in mammoth nuclei. Arrows and asterisks indicate mammoth nucleus-like structures injected and metaphase II chromosomes, respectively. The time (h:min) shown in the images indicates time duration from the starting point of imaging. (**d** and **e**) Representative time-lapse images of γH2A.X (green) before oocyte activation. (**f**) DDI of 14 independent mammoth nuclei 3 h after the NT. Magnified images for nuclei that had the highest and lowest levels of DNA breaks are shown. Note that DNA damage were particularly accumulated at the peripheral region of nuclei. (**g** and **h**) Representative time-lapse image of γH2A.X after activation. (**i**) Time course changes in DDI of mammoth genome in seven independent nuclei. Bar = 10 μm.

Finally, we quantitatively assessed the integrity of mammoth genome and evaluated the feasibility of further development. Extracted genomic DNA from mammoth remains mainly contained fragments of approximately 150–170 bp (Supplementary Fig. S8) and, in particular, muscle samples also showed enrichment of fragments of over 300 bp, whereas those from elephant had a peak only at much longer size. These data indicate that the mammoth genome was highly damaged and, nevertheless, were reminiscent of the presence of nucleosome structure, consisting of histone octamer and 146 bp of DNA in one unit, and in good agreement with the identification of histones by mass spectrometry (Fig. 2c). We subsequently evaluated the integrity of mammoth DNA in living mouse oocytes by monitoring changes in the phosphorylation of histone variant H2A.X (γH2A.X), a marker for DNA double-strand breaks (DSBs)^21^, and assessed the developmental potential of embryos (Supplementary Fig. S9). For reference, we prepared mouse sperm with different extents of DNA damage. Frozen damaged sperm cells showed stronger γH2A.X signals than the fresh ones, whereas H2B was stained as the same level (Fig. 4a). The extent of DNA damage, calculated as γH2A.X/H2B ratio, accurately reflected the actual accumulated level of DNA damage (Fig. 4b). We referred to this γH2A.X/H2B ratio as DNA damage index (DDI) and the median value of fresh sperm was set at 1 (Supplementary Fig. S10). Nuclei carrying DDI more than 2 often failed to support embryonic development after sperm injection (Fig. 4c). In mammoth NT (Supplementary Table S10), some nuclei showed strong γH2A.X signals at 3 h after NT, while others remained weak (Fig. 4d–f and Supplementary Movie S5). Those values were comparable to DDI from frozen-thawed sperm (Fig. 4b and f). After activation, different kinetics of γH2A.X signals were observed (Fig. 4g–i and Supplementary Movie S6) and, surprisingly, DDI almost decreased to the fresh sperm level in some NT oocytes (Fig. 4h and i and Supplementary Movie S6), suggesting that mammoth nuclei potentially stimulate the DNA repair machinery in mouse oocytes. Taken together with Bioanalyzer data (Supplementary Fig. S8), although the presence of nucleus-like structures with DDI comparable to sperm was extremely rare in the Yuka mammoth remains, those ones may have the capability of nuclear remodelling.

In this study, we succeeded in observing the manifestation signs of the biological activity of mammoth nuclei over 28,000 years old in mouse oocytes, such as the histone incorporation, the spindle assembly and the partial pronuclear formation, although the reconstructed oocytes did not show any further cleavage, possibly due to the extensive DNA damage in the transferred nuclei. These achievements could not be made without our unique approach comprising the screening of nucleus-like structures in preserved remains, NT and less-invasive live-cell imaging. This approach, taking advantage of oocyte machinery, may provide the possibility that further nuclear functions, i.e. DNA replication and transcription, can be induced to extinct mammoth nuclei and promote the embryo development with lesser-damaged nuclei. The induction of the DNA repair in mouse oocytes can be another way to promote development. In conclusion, although the results presented here clearly show us again the *de facto* impossibility to clone the mammoth by current NT technology, our approach paves the way for evaluating the biological activities of nuclei in extinct animal species.

## Methods

### Animals

Mouse care and experimental procedures were approved by the Animal Care and Use Committee of Kindai University (Application number: KAAT-22-001). All animal experiments were performed in accordance with the Guide for the Care and Use of Laboratory Animals. Mice were maintained on a 12:12 dark: light cycle at a constant temperature of 22–23 °C and euthanised with cervical dislocation immediately before oocyte collection. Muscle tissues of a female Asian elephant (*Elephas maximus*), named Haruko, were collected from a carcass at necropsy at Osaka Municipal Tennoji Zoological Gardens, and frozen until use. The animal welfare and ethics were in accordance with guidelines adopted by the Japanese Association of Zoos and Aquariums.

### Dating of the Yuka mammoth

The dating was performed using tandem accelerator mass spectrometry (AMS) at the National Institute of Environmental Studies. Briefly, a frozen skin tissue derived from the Yuka mammoth was freeze-dried, homogenised and burned out at 850 °C for 8 h with a piece of copper and reduced copper in a vacuumed silica tube to produce CO_2_. Produced CO_2_ was separated from water by ethanol-dry ice trap (−80 °C) in a vacuumed line. Next, CO_2_ was reduced with H_2_ via iron-catalysed reaction for 8 h and graphitised. The mixed graphite-iron powder was pressed into an aluminium cathode holder and analysed by AMS (NIES-TERRA, Tsukuba, Japan). The 14 C/C ratio was analysed as the percentage of Modern Carbon (pMC) based on the guaranteed value of HOXII standard value issued by the National Institute of Standards and Technology. This 14 C/C ratio was converted to conventional radiocarbon age. Finally, this conventional radiocarbon age was converted to calendar era using Intcal 13.

### Protein extraction and trypsin digestion

Protein extraction from the mammoth tissues and following sample preparation were performed in a laboratory where modern elephantids have never been introduced. To avoid contamination, suits, gloves and facemasks were constantly used. During the procedures, protein investigations of modern animal species were not performed in the laboratory. Muscle samples from an Asian elephant were introduced into the laboratory after the LC-MS/MS measurements of the mammoth samples were completed.

The skeletal muscle and bone marrow tissues (100 mg) were homogenised on ice in 300 μl lysis buffer comprising 7 M urea, 2 M thiourea, 4% (w/v) CHAPS, 0.05% (v/v) tri-n-butylphosphine and protease inhibitor cocktail (Roche Diagnostics) using Sample Grinding Kit (GE Healthcare). The homogenates were centrifuged at 17,000 *g* for 10 min at 4 °C, and the supernatants were transferred to new tubes. This centrifugation step was performed twice to completely remove the tissue debris. Protein concentrations of the supernatants were measured by Bradford assay using γ-globulin as a standard. The protein solutions containing 300 μg proteins were transferred to new tubes, and six-times volume of ice-cold acetone was added to the tubes. After incubation at −20 °C for 2 h, proteins were precipitated by centrifugation at 17,000 *g* for 10 min at 4 °C and dried *in vacuo*. The precipitated proteins were dissolved in 100 μl of 8 M Urea in 200 mM triethylammonium bicarbonate (TEAB) solution. To reduce disulfide bonds, 10 μl of 50 mM Tris-(2-carboxyethyl) phosphine was added to the samples, followed by incubation at 37 °C for 2 h. Next, 5 μl of 200 mM methylmethanethiosulfonate in isopropanol was added to the samples, followed by incubation at room temperature for 30 min to block sulfhydryl residues of cysteines. Four hundred μl of digestion buffer (300 mM TEAB containing 1 mM CaCl_2_) and 30 μl of sequence grade modified porcine trypsin (Roche Diagnostics) dissolved in the digestion buffer at 0.5 μg/μl were added to the samples, followed by incubation at 37 °C for 2 h. Subsequently, 50 μl of double distilled water and 30 μl of the trypsin solution were added to the samples, followed by incubation at 37 °C for 16 h. The reaction was stopped by adding 50 μl of 10% phosphoric acid and 1 ml of 0.1% formic acid. The resulting peptides were desalted with SepPak C18 columns (Merck Millipore) and vacuum-dried.

### LC-MS/MS

Nano LC-MS/MS measurements were performed using a TripleTOF 5600 plus system (AB SCIEX) equipped with NanoSpray III ion source (AB SCIEX) and Eksigent nanoLC system (Eksigent Technologies). An ion-spray voltage of 2300 V, curtain gas of 20 psi, nebulizer gas of 20 psi, an interface heater temperature of 150 °C and Decluster Potential of 80 were applied. MS scans were performed for 0.25 s in the m/z range of 400–1250. Overall, 20 product ions were selected in each MS scan for subsequent MS/MS scan if they exceeded a threshold of 150 counts per sec (counts/s). To minimise repeated scanning, previously scanned ions were excluded for 14 s. Each MS/MS scan was performed for 100 ms at the m/z range of 100–1600. A sweeping collision energy setting of 35 ± 5 eV was applied for all precursor ions. A ChromXP C18-CL 3 μM 120 A, 75 μm × 150 mm capillary column was connected to a Picochip emitter (New Objective), at which the spray voltage was applied. The flow rate was set at 300 nl/min. Mobile phases comprised (A) 0.1% formic acid (FA) and (B) 0.1% FA in 100% acetonitrile. A three-step linear gradient of 2–5% B in 5 min, 5–20% B in 95 min, 20–32% B in 20 min, 90% B for 5 min was employed. Peptide samples were re-suspended in 0.1% FA and 2% acetonitrile at the final concentration of 1.0 μg/μl and 1, 3 and 6 μl of the solution were injected. Blank runs were inserted between the sample runs to avoid potential carryover contamination.

### Database search for identification of proteins and post-translational modifications

Identification of mammoth tissue proteins was performed using Proteinpilot 4.5 software (AB SCIEX). The raw MS spectrometry data was processed using Paragon^TM^ algorithm in the ProteinPilot software to search against UniProt *Mammuthus primigenius* database (134 entries) first and subsequently against UniProt mammalian database (1,375,125 entries), which contains 25,876 proteins of *Loxodonta africana*, 223 of *Elephas maximus*, and 134 of *Mammuthus primigenius*. Trypsin was selected as the digestion enzyme and methane-thiosulfonation as the cysteine alkylation method. All combinations (380 patterns) of one amino acid substitutions and all the described modifications (295) were taken into consideration. False discovery rate (FDR) analyses were performed using a reverse decoy database to reduce false positive hits. Proteins with FDR rates under 1% were considered as significantly identified proteins in this study. Protein-Pilot automatically clustered the identified proteins into groups that share common peptides so that the minimal set of justifiable identified proteins was listed. The protein within each group that can explain more spectral data with confidence is shown in the results as the primary protein of the group. Common potential contaminant proteins such as trypsin and keratins were rejected from the identified protein lists. For annotation analysis, each identified protein was replaced with the homologous human protein using Blast search against UniProt human protein database, and a set of the homologous human proteins was subjected to the database for Annotation, Visualization and Integrated Discovery (DAVID) analysis (http://david.abcc.ncifcrf.gov/).

Comprehensive identification of post-translationally modified peptides was also performed using Proteinpilot 4.5 software. To enhance confidence of the identification, database search was performed under more restricted conditions. Specifically, the raw MS spectrometry data was searched against UniProt database taxonomically restricted to *L. Africana*. Amino acid substitutions were not taken into consideration. Peptides identified with confidence lower than 95% were rejected. The other parameters or data processing were the same as those of the protein identification.

### Estimation of post-mortem protein damage

The extent of non-enzymatic deamidation of glutamine and asparagine is related to time and environmental conditions^22^ and potentially useful as a marker of bone collagen deterioration^16^. To compare preservation states of our samples between the tissues and with those of the previously reported mammoth remains^8,17^, we checked all the identified collagen sequences with various modifications one by one and calculated the deamidation rates according to the methods of Orlando *et al*.^17^. We counted a sequence as deamidated when a deamidated version of the sequence was identified even once.

Quantitative analyses of the collagen peptides were performed using PeakView 2.2 software (AB SCIEX). Extracted ion chromatograms (XIC) were generated for non-deamidated and amidated forms of five collagen peptides, COL1A1-0912-0 (GPAGPQGPR), COL1A1-0510-1 (GVQGPPGPAGPR with oxidation at P6), COL1A1-0441-0 (DGEAGAQGPPGPAGPAGER), COL1A1-0441-1 (DGEAGAQGPPGPAGPAGER with oxidation at P10) and COL1A1-0939-3 (GFSGLQGPPGPPGSPGEQGPSGASGPAGPR with oxidation at P9, P12 and P15), whose deamidation rates were reportedly correlated with the thermal age of the collagen^16^. Although a deamidated form of a peptide (+0.984 Da) shows almost the same m/z value as one of isotopic variants of the non-deamidated form (+1.0 Da from a monoisotopic peak); the two peptides can be separately quantified because of their different retention times.

### Whole-genome sequencing

Sample preparation and DNA extraction were performed in a dedicated ancient DNA laboratory into which modern elephantids have never been introduced. To avoid contamination, laboratory bench surfaces and tools were frequently sterilised by bleaching or UV-irradiation and suits, gloves and facemasks were constantly used during all steps. The Yuka mammoth’s genomic DNA derived from skeletal muscle tissues (50 mg) or bone marrow tissues (50 mg) were purified and collected using QIAGEN DNeasy Blood & Tissue Kit (69504, QIAGEN). DNA samples of 3.7 μg and 1.2 μg were collected from the skeletal muscle tissue and the bone marrow tissue, respectively. Both DNA samples were resuspended in TE (pH 8.0) and preserved at 4 °C. Cytosine deamination is a major post-mortem damage of DNA. The resulting product (uracil) directs the incorporation of adenine during library preparation, which eventually results in C-to-T or G-to-A substitutions, particularly in terminal regions of DNA fragments. To exclude this type of DNA lesions, we constructed Illumina DNA libraries using NEB next DNA Sample Prep Master Mix containing Phusion polymerase (New England Biolabs), which is unable to replicate through uracil^23^. Quality and quantity of the derived libraries were assessed by 2100 Bioanalyzer using DNA 1000 kit (Agilent Technologies) and qPCR with KAPA library Quantification Kit (Kapa Biosystems), respectively. After the cluster generation on the Illumina cBot (Illumina), sequencing was performed on Hiseq. 2500 (Illumina).

The same mammoth DNA samples, each of 10 ng, and DNA from Asian elephant were analysed by 2100 Bioanalyzer using High sensitivity DNA kit (Agilent Technologies).

### Bioinformatic analysis

Obtained sequence data of 100 bp (paired end), 102 bp and 50 bp (single read) lengths were filtered to remove PCR duplicates, reads with low base quality score (QV < 20), reads containing ambiguous nucleotide ‘N’ and the sequences from phix control library (Illumina), which were spiked into each lane. Adapter sequence was also trimmed. Quality filtered reads were used for the alignment against the African elephant genome sequence (loxAfr3) by BWA (ver. 0.7.5a)^24^ with default settings. After alignment, sequence reads aligned uniquely and properly (for paired end) to the reference genome were used for SNP calling, which was performed using SAMtools (ver. 0.1.19)^25^ with default settings except for adding following criteria: minimum read depth = 3 and minimum SNP = 2. Identified SNPs were annotated according to the gene sets downloaded from the Ensembl website [http://www.ensembl.org/Loxodonta_africana/Info/Index]. First, they were classified into intergenic or genic regions. Those among genic regions were further separated into exons (synonymous or non-synonymous) and introns. Insertions/deletions were also identified as described above. They were classified into intergenic and genic (intron or CDS) regions. Consensus sequence was reconstructed using SAMtools and custom perl script. To infer the sequence of mitochondrial genome, using Bowtie2^26^ with the default option, we aligned the filtered reads of a sequence run (KM2_SR100) to the known mammoth M19 mitochondrial genome (EU153448), the end of which was added to the first 100 bp to enable alignment effectively, resulting in the generation of an average 346-fold coverage over the entire length. We used sequence reads that aligned to mitochondrial DNA to assess the sequence substitution by comparing the individual reads with their consensus. The number of different or common bases was calculated in each site. DNA damage was assessed by investigating substitution patterns, which have been found to be elevated at 5′- and 3′-ends using mapDamage 2.0^27^.

### Phylogenetic analysis

The complete mitochondrial sequences from the Yuka mammoth, 19 other woolly mammoths and 2 extant elephants and mastodon, used as an outgroup, were used for phylogenetic analysis. The mitochondrial sequences were aligned using MUSCLE and the resulting multiple alignments were subjected to the construction of a phylogenetic tree by maximum likelihood using MEGA6^28^.

### Preparation of mammoth nuclei for nuclear transfer

Buffer A was prepared by mixing 98 μl of the nuclear isolation media (731086; Beckman Coulter) and 2 μl of 0.05% trypsin-EDTA solution (25300-054; Thermo Fisher Scientific). Mammoth muscle tissue (approximately 30 mg) was minced with forceps and scissors in a culture dish. Next, the minced muscle tissue was transferred into a BioMasher column, followed by the addition of 100 μl Buffer A and homogenisation using the BioMasher III (320302; Nippi). After centrifugation at 900 *g* for 4 min, the supernatant was transferred into a new 0.2 ml tube, the same volume of TE buffer (pH 8.0) was added and mixed well, and the mixed solution was centrifuged at 400 *g* for 4 min. The supernatant was removed and the pellet was suspended into 30 μl of CZB medium. DAPI (D3571; Thermo Fisher Scientific) was added to the nuclei suspension at a final concentration of 0.1 µg/ml. The suspension was transferred into a new culture dish and somatic cell nuclei with intrinsic fluorescence were picked up under a fluorescent microscope for nuclear transfer.

### γH2A.X monoclonal antibody

To generate monoclonal antibodies directed against γH2A.X, serine 139-phosphorylated form of histone H2A.X, mice were immunised with a synthetic peptide 163-6 [CGGKKATQA(phospho-S)QEY] as described previously^29^. After generating hybridomas, clones were screened by ELISA using peptides; 163-5 [CGGKKATQASQEY], 163-6 [CGGKKATQA(phospho-S)QEY], 163-7 [CGGKKA(phopho-T)QASQEY], 163-8 [CGGKKA(phopho-T)QA(phospho-S)QEY] and H3S10P [ARTKQTARK(phospho-S)TGGKAPRKQC]. Clone CMA281 specifically reacted with the peptides containing phospho-S139 and isotyped as IgG1-κ using IsoStrip Mouse Monoclonal Antibody Isotyping Kit (Roch). Antibody purification, Fab preparation and fluorescent dye conjugation were performed as described previously^30^. To validate the reactivity of CMA281 to cellular γH2AX in a damage-dependent manner, HeLa cells were untreated or treated with etopside (20 μg/ml; 20 min) and stained with purified IgG followed by Alexa Fluor 488-conjugated donkey anti-mouse IgG (Jackson Immunoresearch) and Alexa Fluor 488-labelled Fab. DNA was counterstained with Hoechst33342^30^.

### Immunofluorescence

Immunofluorescence of cell nuclei was performed using a micromanipulator system (Fig. S5). Individual nucleus-like structures were fixed with 3.7% paraformaldehyde (w/v) in phosphate buffered saline (PBS) for 15 min and permeabilised with 2% Triton X-100 in PBS for 2 h at room temperature. After fixation and permeabilisation, the samples were incubated in the blocking buffer (3% BSA + 0.02% Tween in PBS) for 1 h at room temperature and incubated with primary antibodies against histone H3 (1:50) and lamin B2 (1:50) in blocking buffer at 4 °C overnight. The samples were washed with blocking buffer for 15 min at room temperature; this step was performed three times. The samples were then incubated with secondary antibodies (1:200) for 1 h at room temperature. The DNA was stained with 0.05 µg/ml of 4′, 6-diamidino-2-phenylindole (Thermo Fisher Scientific., D3571) in blocking buffer for 1 min. Finally, the samples were mounted on a slide glass and examined with a laser-scanning confocal microscope (Zeiss LSM800 Axio Observer Z1) and imaging software (Zeiss ZEN 2).

### Nuclear transfer (NT)

NT was performed as described previously^31^. Metaphase II (MII) oocytes were collected from B6D2F1 female mice (Japan SLC). Briefly, nuclei of the donor muscle tissue (DAPI positive nuclei) were injected into MII oocytes using Piezo Micro Manipulator (Prime Tech). After NT, morphologically normal oocytes were incubated for 0.5–1 h in CZB medium before live-cell imaging analysis. After 3 h of NT, the reconstructed oocytes were chemically activated by 5 mM SrCl_2_ and 2 mM EGTA containing KSOM medium in presence of 5 µg/ml cytochalasin B for 6 h^31^.

### Live-cell imaging

Synthesis and purification of mRNA for injection have been described previously^32^. MII oocytes collected from B6D2F1 mice (10–20 weeks old) were injected with a mixture of 10 μg/ml histone H2B-mCherry mRNA and 10 μg/ml EGFP-EB1 mRNA^20^ using Piezo Micro Manipulator to visualize DNA and microtubule. For measurement of DNA double-strand break, a mixture of 10 μg/ml histone H2B-mCherry mRNA and 0.2 mg/ml of Alexa488-labled anti-γH2A.X Fab fragment was injected. The γH2A.X is phosphorylated form of histone H2A variant H2A.X at serine 139 and a marker for DNA DSBs^21^. It was used for the quantification of DNA integrity based on the FabLEM technology^30^ in which the kinetics of protein modification could be captured in living cell^32^. H2B-mCherry was used for the normalization of injection volume in each embryo. After 4–5 h of incubation, the oocytes were injected with somatic cell nuclei and transferred to drops of KSOM medium on a glass-bottom dish (MatTek) and placed in an incubation chamber stage (Tokai Hit) at 37 °C under 6% CO_2_, 5% O_2_ and 89% N_2_ atmosphere on an inverted microscope (IX-71; Olympus), which was equipped with a spinning disk confocal system (CSU-W1; Yokogawa Electric), EM-CCD (iXon3-888; Andor Technology), plus a filter wheel and z-motor (Mac5000, Ludl Electronic), in a temperature-controlled dark room at 30 °C. Two-colour fluorescence images in 51 different focal planes with 2 μm intervals were captured every 4–5 min using UPLSAPO30XS (NA = 1.05) or UPLSAPO60XS (NA = 1.30) objective lens with 488- (Melles Griot) and 561-nm (Cobolt) laser lines employing MetaMorph software ver. 7.7.10 (Molecular Devices). Image editing was performed using the same MetaMorph software. Image processing and three-dimensional measurements of signal intensities of histone H2B and γH2A.X were performed using Volocity software ver. 6.3 (Perkin Elmer). Scheme for the image analysis and definition of DNA Damage Index (DDI) are summarised in Fig. S10. Normality and homoscedasticity of DDI in each sperm sample were examined by Shapiro–Wilk test and Bartlett test, respectively. After these analyses, significances with control (fresh sperm) were analysed by Steel test.

### Sperm preparations and Intracytoplasmic Sperm Injection (ICSI)

For preparation of frozen-thawed sperm samples, cauda epididymal sperm were collected from B6D2F1 mice (12–24 weeks old), suspended in Hepes-CZB medium (2.0 × 10^7^ sperm/ml) and frozen at −30 °C in a refrigerator. For DNase treated sperm, cauda epididymal sperm were suspended in DNase buffer containing 50 mM Tris-HCl (pH 7.5), 10 mM MnCl_2_/4H_2_O and 100 mM NaCl at the same sperm concentration (2.0 × 10^7^ sperm/ml), and DNase (RQ1 RNase-free DNase, M6101, Promega) was added at the final concentration of 50 U/ml. Sperm suspensions were incubated at 37 °C for 15 min; subsequently, DNase was inactivated by incubation at 65 °C for 10 min. One microliter of each sperm suspension was mixed with 9 µl of Hepes-CZB medium containing 12% (w/v) polyvinylpyrrolidone (PVP) and was applied to intracytoplasmic sperm injection (ICSI). Protocols for ICSI have been described elsewhere. Briefly, the head of each sperm was separated from the tail by applying Piezo pulses to the head–tail junction using piezo-driven pipette. Only the sperm head was injected into each MII oocyte cytoplasm. After ICSI, the fertilised embryos were live-cell imaged, as described above, and further cultured in KSOM medium at 37 °C under 6% CO_2_, 5% O_2_ and 89% N_2_ until the blastocyst stage.

### ***In vitro*** fertilisation and embryo transfer

Cumulus-intact oocytes were collected in 0.2 ml of TYH medium and inseminated with capacitated sperm (final concentration, 100 sperm/μl). After 2 h of incubation at 37 °C under 5% CO_2_ in air, cumulus cells were dispersed by brief treatment with hyaluronidase (Type-IS, 150 units/ml, Sigma). The embryos immediately after fertilisation were injected with the mixture of histone H2B mRNA and γH2A.X Fab fragment as described above and further cultured in KSOM. Two-cell stage embryos were transferred into the oviduct of surrogate mothers (ICR strain) on Day 1 of pseudo-pregnancy following mating with vasectomised ICR males.

## Supplementary information

Supplementary Materials

Time-lapse image of histone H2B-mCherry (H2B, red) and EGFP-EB1 (EB1, green) dynamics in oocytes immediately after the injection of elephant (A) and mammoth (B and C) nuclei.

Time-lapse image of nuclear formation processes after the activation of NT oocytes reconstructed with an elephant or mammoth somatic nucleus.

Time-lapse image of chromosome (H2B, red) and spindle (EB1, green) dynamics in an oocyte immediately after the injection of mammoth nucleus.

Time-lapse image of mammoth chromosome (H2B, red) and spindle (EB1, green) dynamics immediately after the activation of the same oocyte with the Supplementary Movie 5.

Kinetics of DSBs in a mammoth nucleus immediately after injection into mouse oocyte.

Kinetics of DSBs in the mammoth nucleus after oocyte activation.

List of proteins identified in 1) the mammoth bone marrow and 2) the mammoth muscle.

List of the GO terms enriched in the 1) bone marrow and 2) muscle samples.

Compatible amino acid substitutions identified in the woolly mammoth by genomic and proteomic analyses.

Summary of comprehensive analysis of post-translationally modified peptides.

## Data Availability

Nucleotide sequence data reported are available in the DDBJ database under the Accession Numbers DRX053291-DRX053293 and LC136999. The mass spectrometry proteomics data have been deposited to the ProteomeXchange Consortium via the PRIDE^33^ partner repositry with the dataset identifier PXD012741.
