# Supplementary material for: Signs of biological activities of 28,000-year-old mammoth nuclei in mouse oocytes visualized by live-cell imaging
Source: Sci Rep. 2019 Mar 11;9:4050. doi: 10.1038/s41598-019-40546-1 (PMC6411884; doi:10.1038/s41598-019-40546-1)
Supplement: Supplementary file 1 — Supplementary Materials [file 41598_2019_40546_MOESM1_ESM.docx]

Supplementary Materials for

**Signs of biological activities of 28,000-year-old mammoth nuclei in mouse oocytes visualized by live-cell imaging**

Kazuo Yamagata†, Kouhei Nagai†, Hiroshi Miyamoto†, Masayuki Anzai†, Hiromi Kato†, Kei Miyamoto, Satoshi Kurosaka, Rika Azuma, Igor I. Kolodeznikov, Albert V. Protopopov, Valerii V. Plotnikov, Hisato Kobayashi, Ryouka Kawahara-Miki, Tomohiro Kono, Masao Uchida, Yasuyuki Shibata, Tetsuya Handa, Hiroshi Kimura, Yoshihiko Hosoi, Tasuku Mitani, Kazuya Matsumoto, Akira Iritani*

**†These authors contributed equally to this work**.

*Correspondence to: iritani@waka.kindai.ac.jp

**This file includes:**

Supplementary Figures S1–S10

Supplementary Tables S1, S6–S10

References (22–32)

Captions for Supplementary Tables S2– S5

Captions for Supplementary Movies S1–S6

**Other Supplementary Materials for this manuscript includes the following:**

Supplementary Tables S2–S5

Supplementary Movies S1–S6


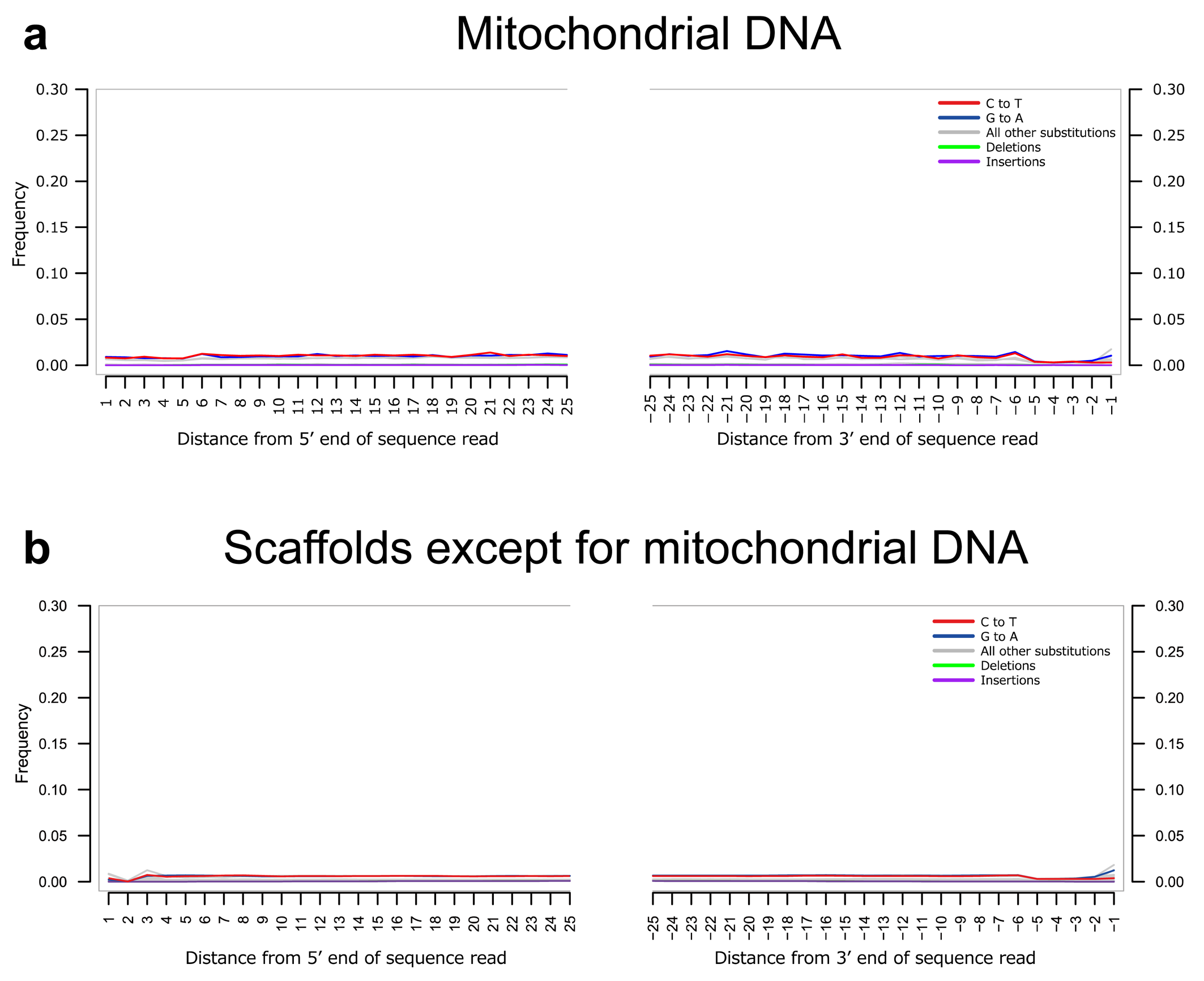


**Fig. S1.** Substitution patterns at the 5′ and 3′ ends of the sequence reads of the Yuka mammoth genome. Frequencies of C-to-T (red) and G-to-A (blue) substitutions are shown for the mitochondrial genome (a) and nuclear genome (b).


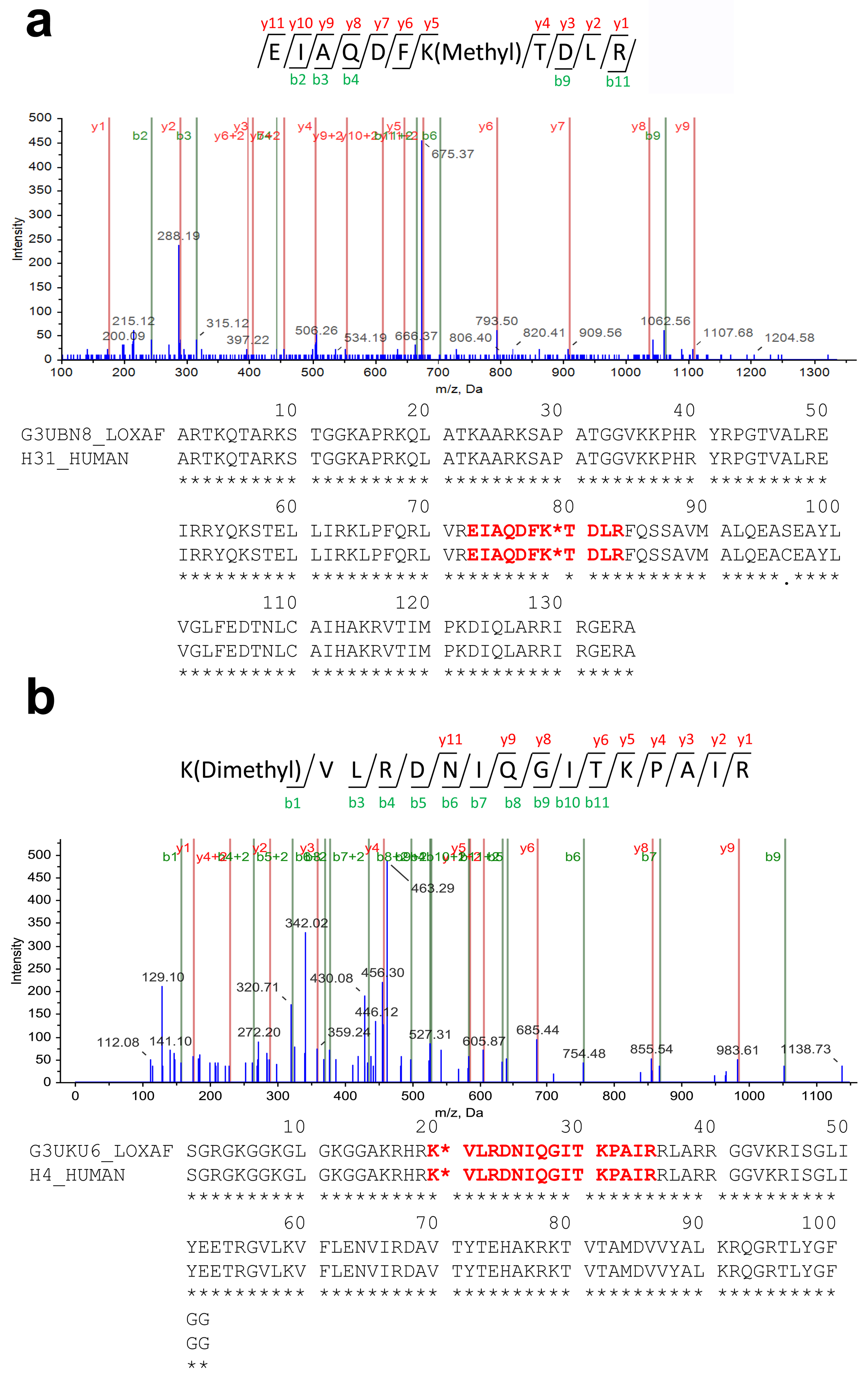


Fig. S2. The detection of histone methylation in the Yuka mammoth. (a) Identification of methylation of histone H3K79. Upper panel: MS/MS spectrum of a doubly-charged precursor ion at m/z = 675.36 was identified as a peptide of EIAQDFKTDLR with methylation at K7. All of the y-series fragment ions (indicated by red lines) and 6 out of the 11 b-series ions (indicated by green lines) were detected, increasing the reliability of the identification; lower panel: The amino acid sequences of histone H3 of *Loxodonta africana* (G3UBN8_LOXAF) and human (H31_HUMAN). The identified sequence and the methylated lysine were consistent with the highly conserved sequence of histone H3 (residues 73–83, indicated by red characters) and an epigenetic modification known as histone H3K79 methylation (indicated by asterisk), respectively. (b) Identification of dimethylation of histone H4K20. Upper panel: MS/MS spectrum of a quadruply charged precursor ion at m/z = 463.29 was identified as a peptide of KVLRDNIQGITKPAIR with dimethylation at K1. Nine out of the 16 y-series ions (indicated by red lines) and 10 out of the 16 b-series ions (indicated by green lines) were detected, increasing the reliability of the identification; lower panel: The amino acid sequences of histone H4 of *L. africana* (G3UKU6_LOXAF) and human (H4_HUMAN). The identified sequence and the methylated lysine were consistent with the highly conserved sequence of histone H4 (residues 20-35, indicated by red characters) and an epigenetic modification known as histone H3K79 methylation, respectively.


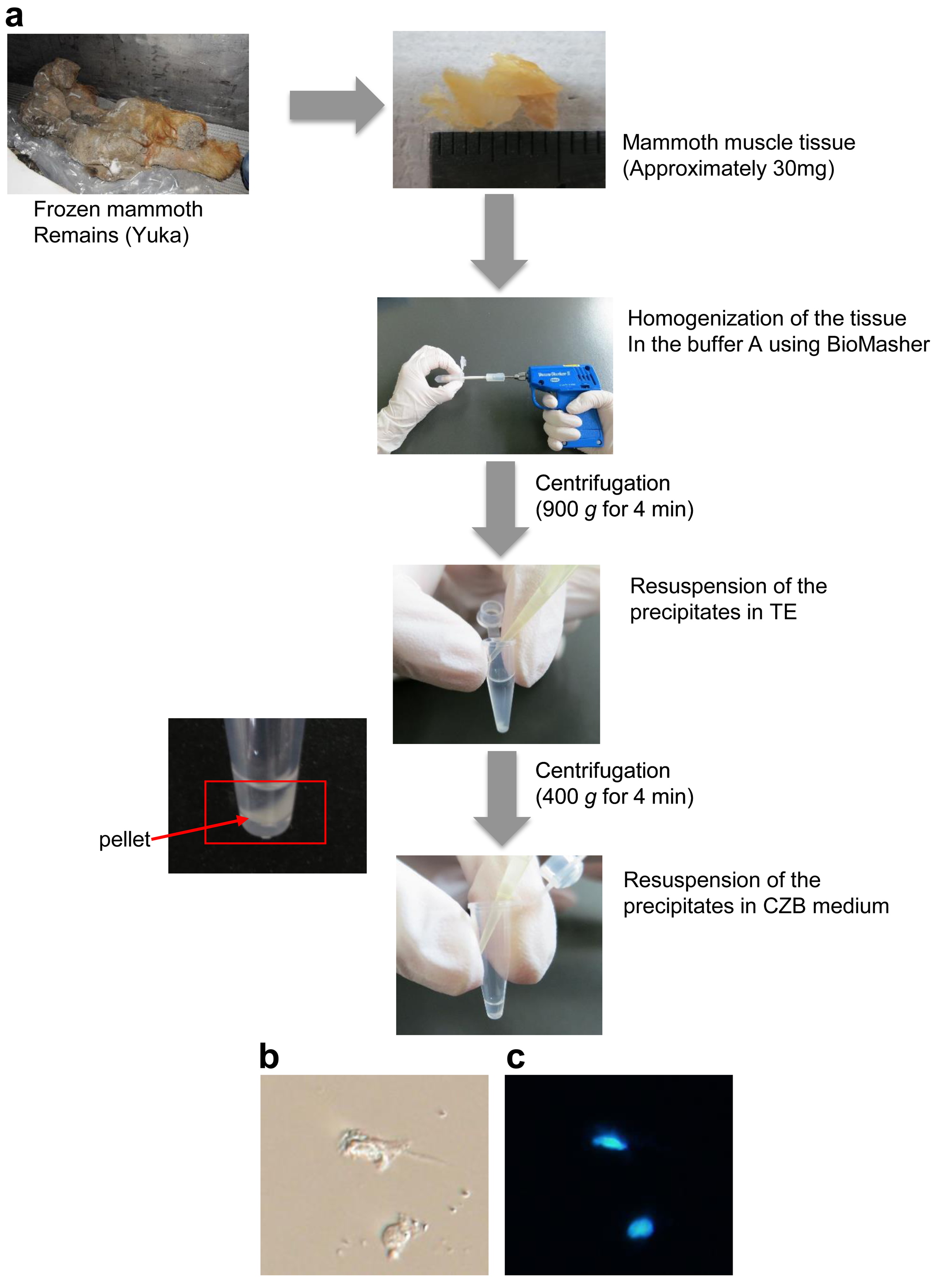


Fig. S3. Preparation of mammoth nuclei from remains for nuclear transfer. (a) Overview of the purification of mammoth nuclei. (b and c) The isolated nucleus-like structures after homogenising the mammoth muscle tissue (b: bright field, c: DAPI staining).


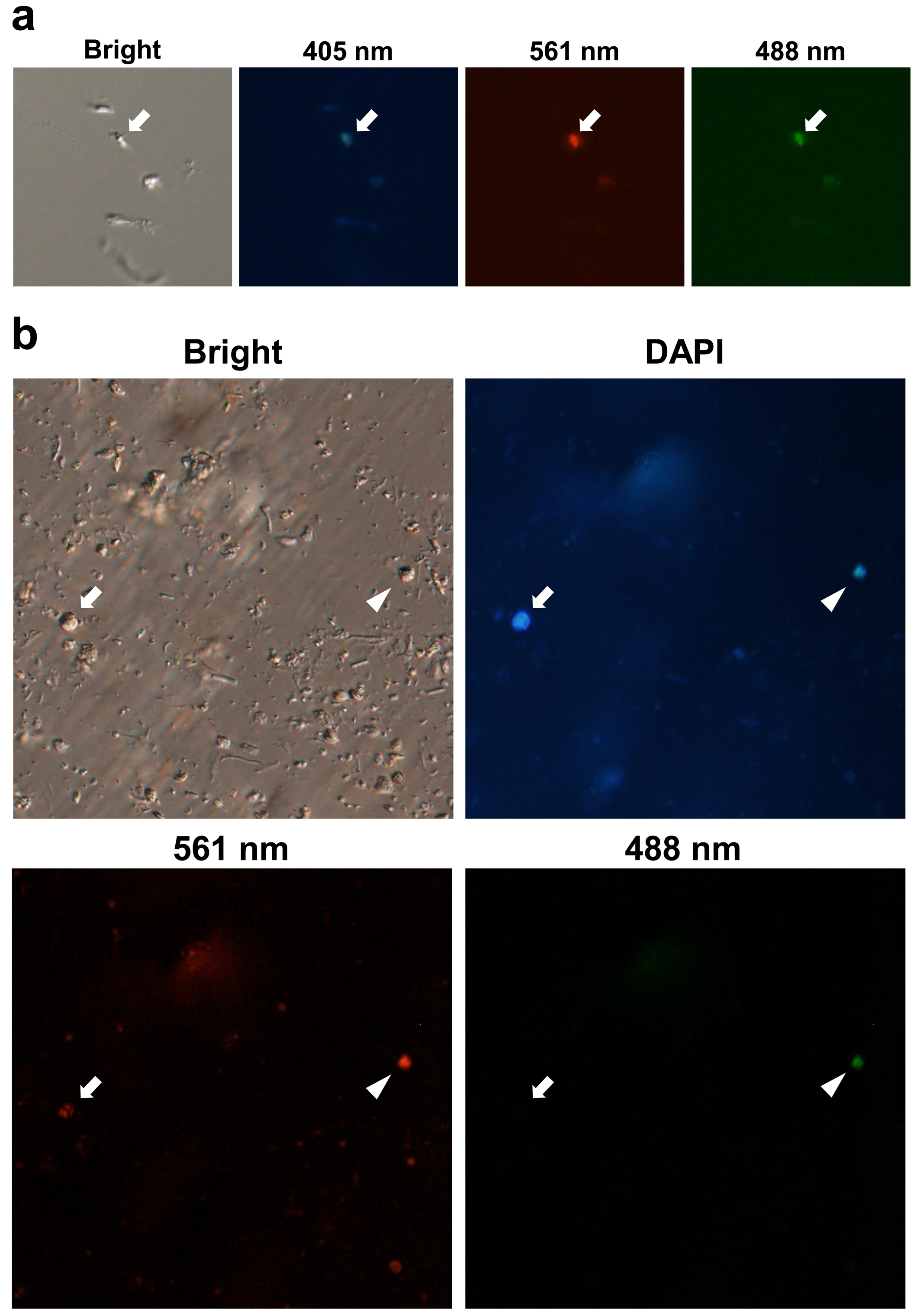


Fig. S4. Selection of nucleus-like structures from the mammoth tissue for nuclear transfer by excluding autofluorescence entities. (a) Fluorescent observation of the homogenates by 405, 488 and 561 nm excitations without any staining. Some structures (arrows) show strong autofluorescence. (b) DAPI-staining of mammoth tissue homogenates. Two nucleus-like structures are positive for DAPI staining. The structure indicated by an arrowhead is positive for both red and green colours, whereas the structure indicated by an arrow is positive only for the red. We chose DAPI-positive and autofluorescence-negative nucleus-like structures as the donor nuclei in subsequent experiments. The conditions for DAPI-staining in this assay have little effect on the nuclear remodeling and embryonic development at least up to the 2-cell stage in somatic nuclear transfer using elephant and mouse muscle cells.


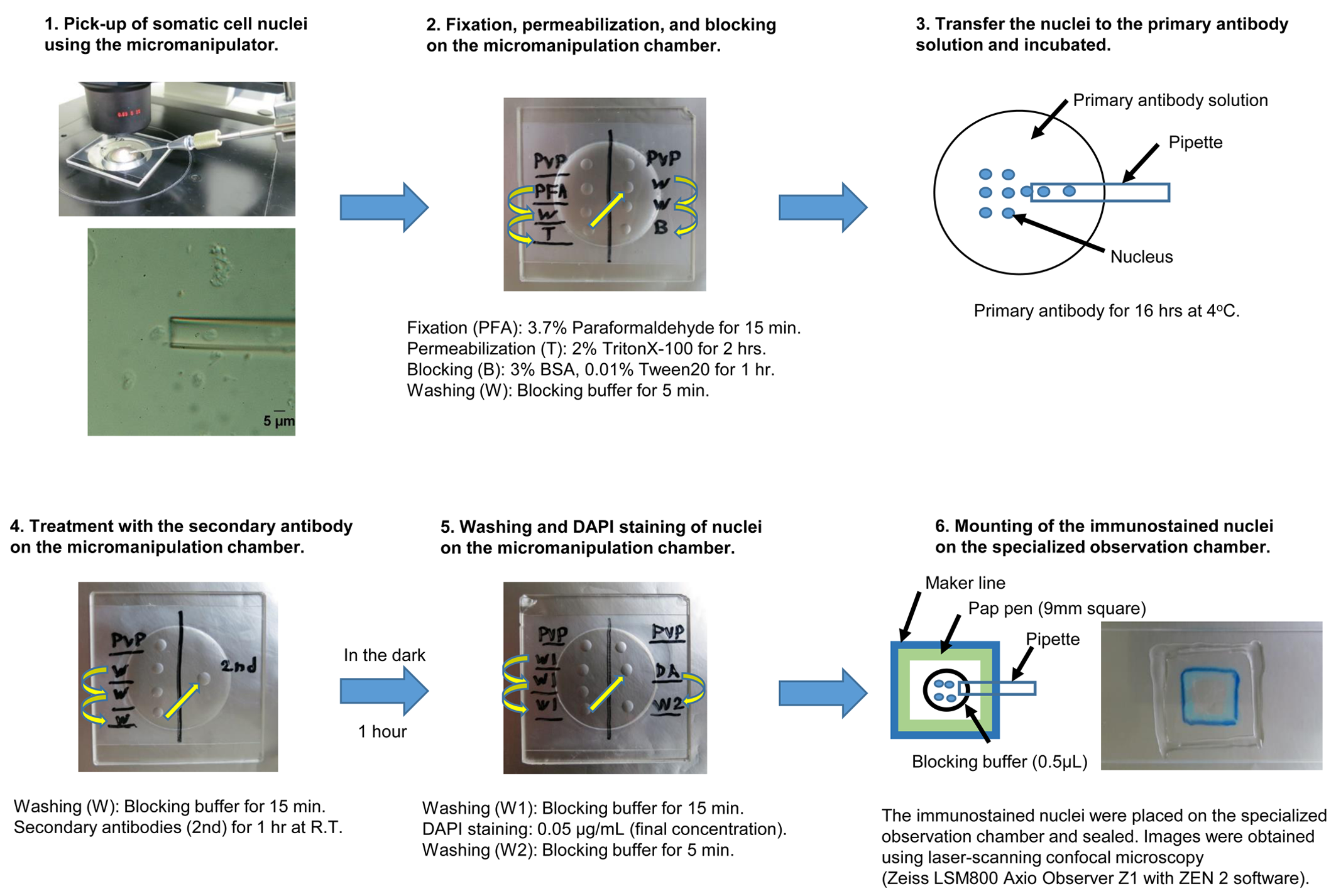


**Fig. S5.** Immunostaining of cell nuclei using micromanipulation system.


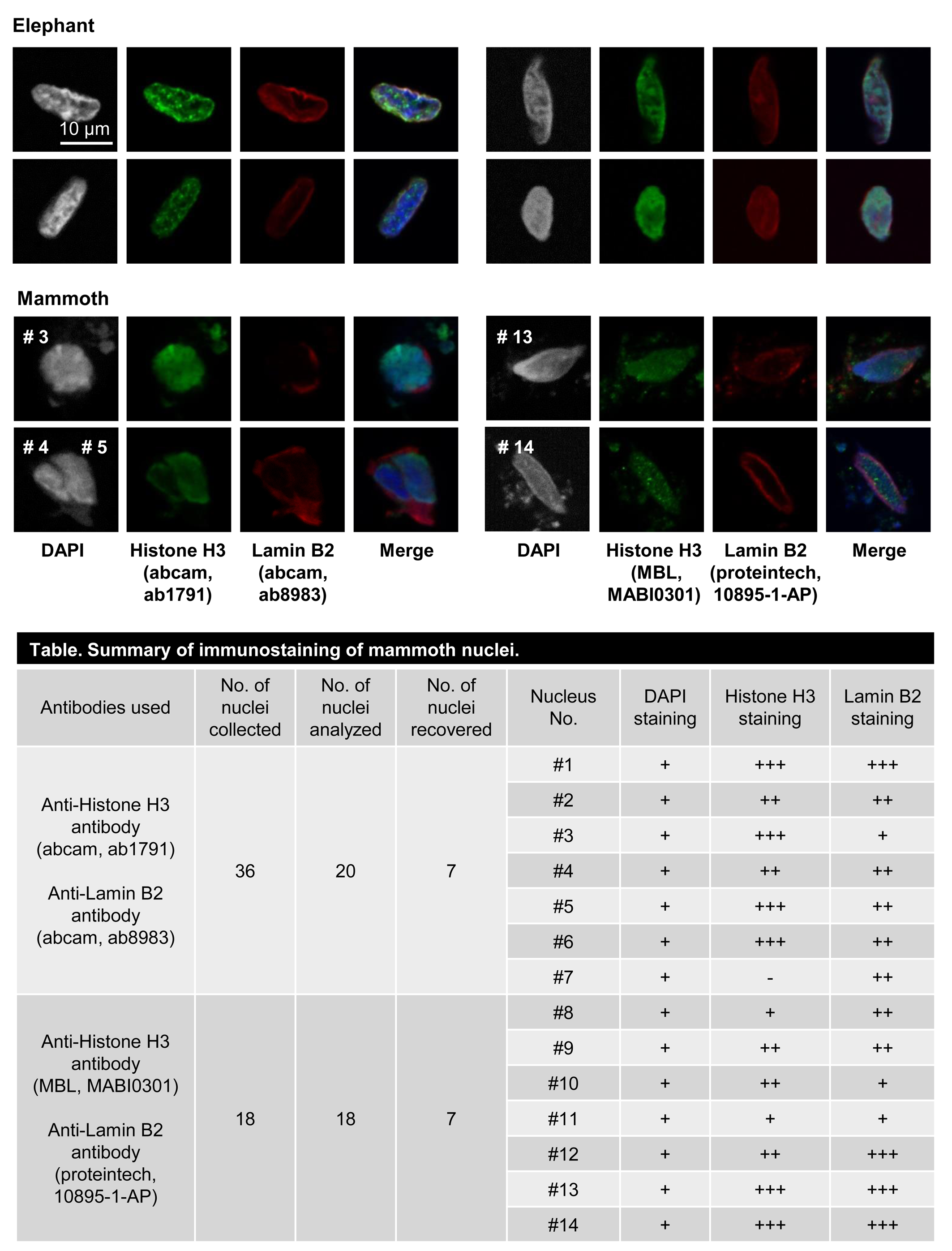


**Fig. S6.** Immunostaining of nucleus-like structures from elephant or mammoth tissues with anti-histone H3 and anti-lamin B2 antibodies. Antibodies obtained from different companies were used and conditions for staining are shown in Supplementary Materials and Methods. Results for staining are summarised in Table. Representative images of each nucleus stained are shown in upper panels. IDs of nuclei correspond to those in Table. DAPI signals are pseudo-coloured in black/white.


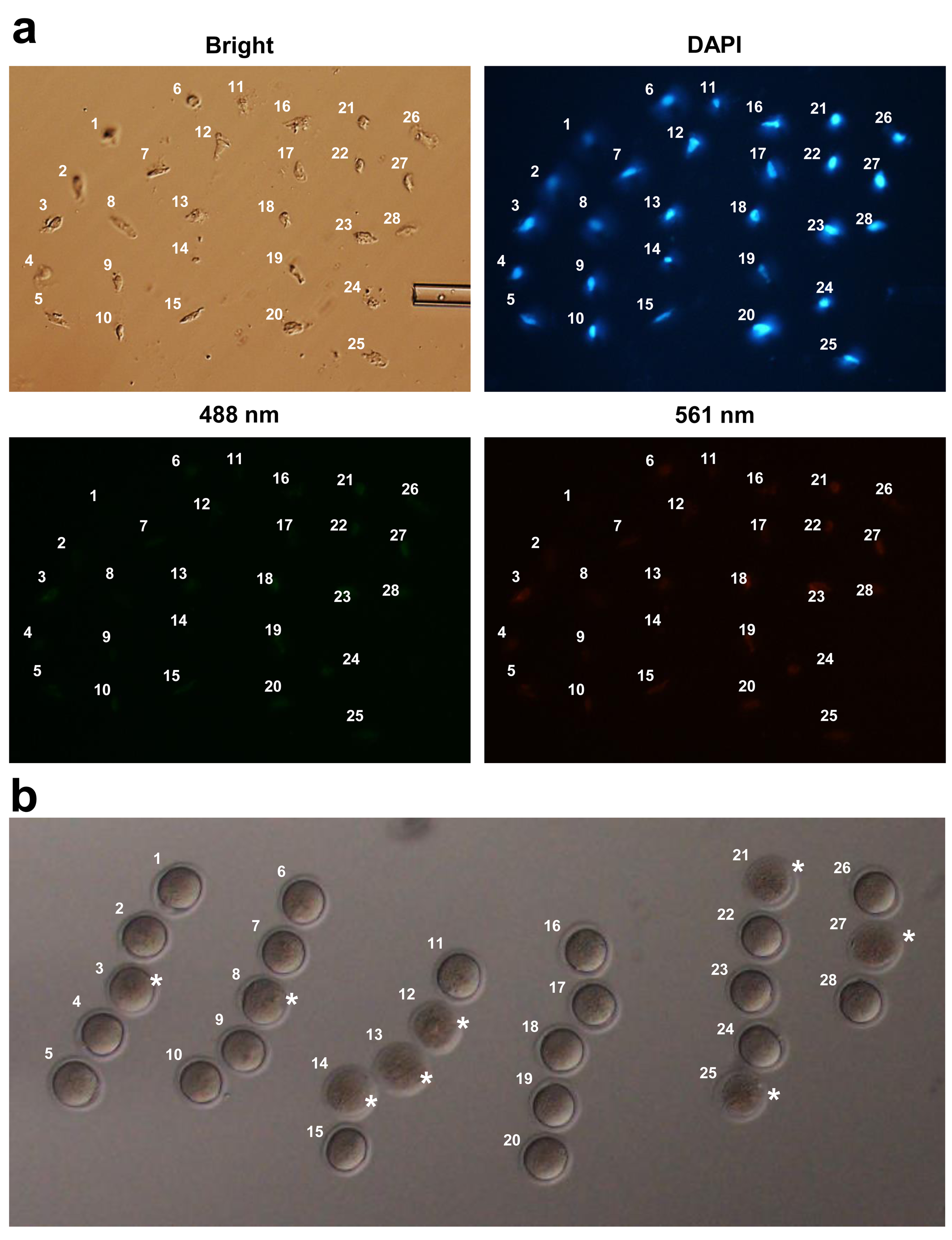


Fig. S7. One-by-one injection of mammoth nucleus-like structures into mouse metaphase II oocytes. (a) Nucleus-like structures collected from frozen mammoth tissues. Twenty-eight structures with DAPI-positive and autofluorescence-negative by 488- and 561-nm excitations are shown. (b) Oocytes injected with mammoth nucleus-like structures and aligned in the same manner as the above panels. Asterisks indicate dead oocytes after injection.


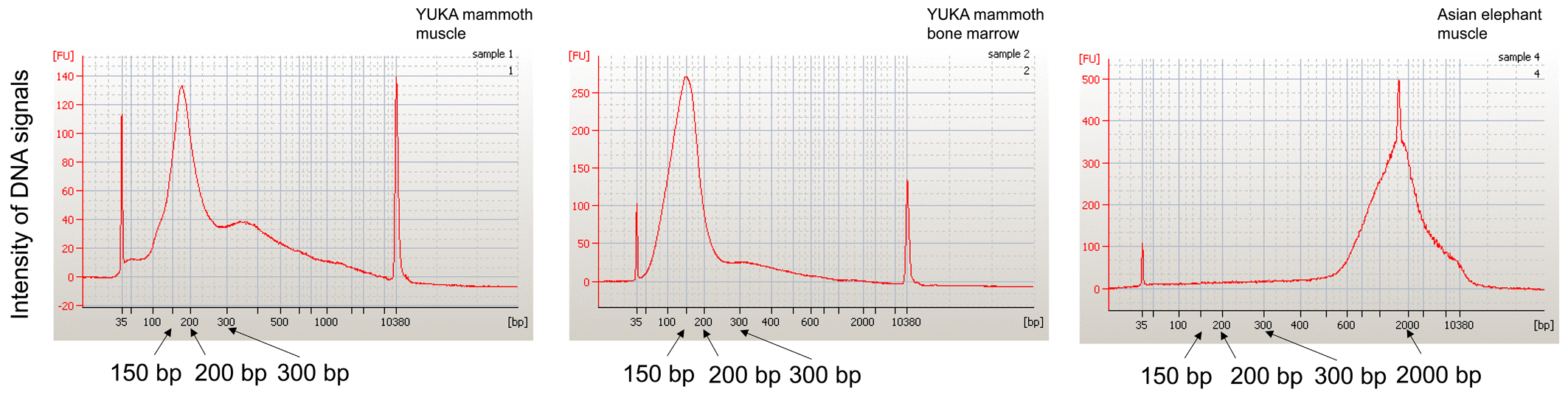


Fig. S8. The size distribution of mammoth and elephant DNA as revealed by bioanalyser. DNA fragments of approximately 150–170 bp are found in mammoth samples. Lower and upper markers are at 35 and 10,380 bp, respectively.

**
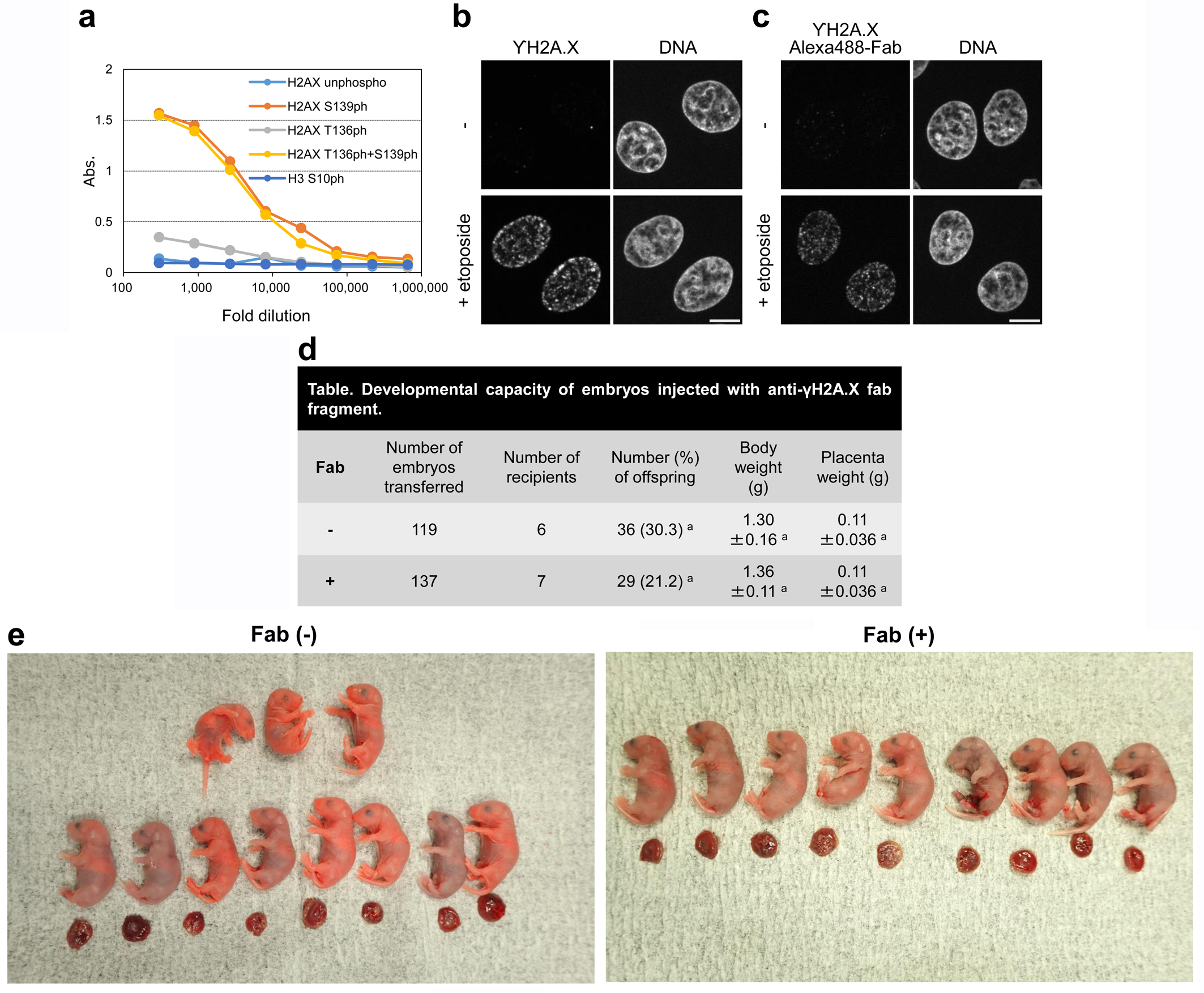
**

**Fig. S9.** Validation of γH2A.X antibody. (a) The culture supernatant of hybridoma clone CMA281 was analysed by ELISA using peptides containing different modifications. Microtiter plates coated with the indicated peptides were incubated with 3-fold dilutions starting from 1:300 dilution. After incubation with peroxidase-conjugated secondary antibody and washing, the colourimetric signal of tetramethylbenzidine was detected by measuring the absorbance at 405 nm (Abs.) using a plate reader. Clone CMA281 reacted specifically with the peptides containing phospho-S139. (b and c) HeLa cells, which were untreated (-) or treated with 20 μg/mL of etoposide for 20 min (+ etoposide), were fixed with 4% paraformaldehyde, immunostained with CMA281 antibody and detected using Alexa Fluor 488 labelled secondary antibody (b) or with Fab fragment of CMA281 antibody labelled with Alexa Fluor 488 (c). DNA was counterstained with Hoechst33342. Scale bars = 10 μm. (d and e) Safety of γH2A.X antibody to the embryonic development. In vitro fertilised pronuclear-stage embryos from B6D2F1 mouse were injected (+) or non-injected (-) with mixtures of 0.1 mg/ml of Alexa488-labelled γH2A.X Fab fragment and 5 ng/μg of histone H2B-mCherry mRNA were cultured until two-cell stage in KSOMaa medium and transferred to the oviduct of pseudo-pregnant females (ICR strain). * Note that there was no significance in full-term development, weights of body and placenta between injection (+) and (-) groups (d, *P* > 0.1, Student’s *t*-test). Pups and placentas from each experimental group are shown in e.

**
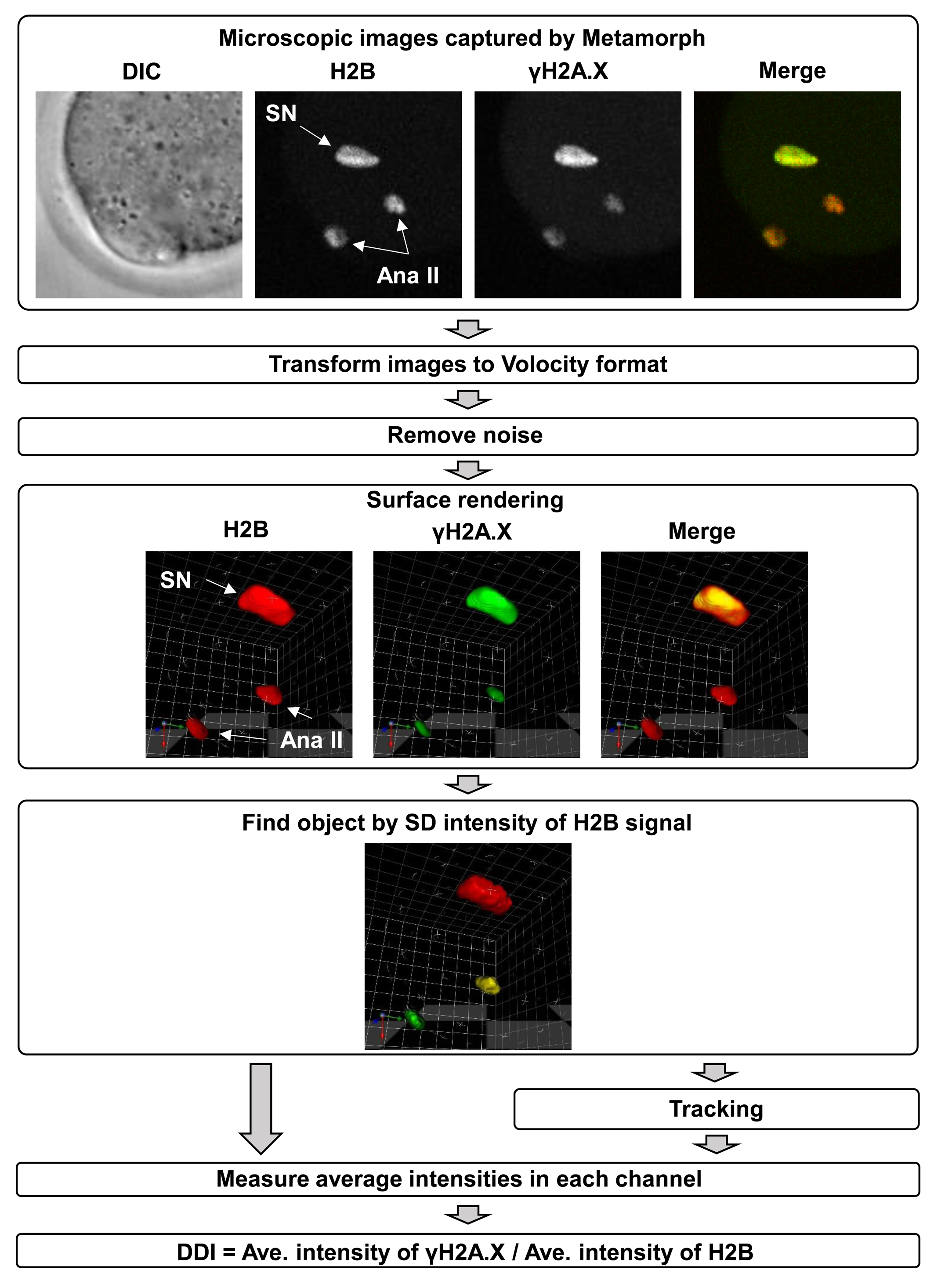
**

**Fig. S10.** Scheme of the image analysis to quantify the DNA damage in nuclei of mouse embryos. For example, the embryo injected with frozen-thawed sperm, also shown in Figure 4A, is indicated. To reduce the influence of background noise, the intensities for γH2A.X and histone H2B fluorescent signals were measured three-dimensionally. Four-dimensional (x, y, z and t) sets of microscopic images (TIFF format) taken by MetaMorph software (Molecular Devices, CA, USA) were transformed to Volocity software (PerkinElmer, MA, USA) format. After removing the noise using median filter (3 x 3 pixel), the nuclei of embryos were visualised three-dimensionally by surface rendering of γH2A.X and histone H2B signals. To define the volume of nuclei, each nucleus was segmented by ‘Find object using SD intensity’ algorithm using histone H2B signal. Average intensities of each channel inside the nuclei defined above were measured; subsequently, γH2A.X/H2B ratio was defined as DNA damage index (DDI).

Table S1: Summary of whole-genome sequencing data.

| Sequence run | Read length | Sequencing | Number of reads | Mapped reads to the African elephant genome (%) | Total mapped sequences (Gb) |
| --- | --- | --- | --- | --- | --- |
| KM2_PE | 100 | PE | 390,159,196 | 51.6 |  |
| KM2_SR50 | 50 | SE | 40,576,383 | 89.4 | 73.9 |
| KM2_SR100 | 102 | SE | 1,015,485,045 | 50.2 |  |
|  |  |  |  |  |  |
| SE and PE represent single-end and paired-end sequencing, respectively. | | | | |  |

**Table S6: Ratio of deamidated collagen peptides in the Yuka mammoth samples compared to previously reported Late Pleistocene mammoth samples.**

|  | Yuka  Muscle | Yuka Bone marrow | Siberian  mammoth | Dent mammoth | La Sena mammoth |
| --- | --- | --- | --- | --- | --- |
| **Unique Collagen Sequences** |  |  |  |  |  |
| Total | 955 | 860 | 172 | 100 | 96 |
| Deamidable (%) | 65.03 | 62.56 | 56.98 | 56.00 | 56.25 |
| Deamidated (%) | 46.22 ^a^ | 54.65 ^b^ | 60.2 ^b^ | 92.86 ^c^ | 88.89 ^c^ |
|  |  |  |  |  |  |
| **Modified Collagen Sequences** |  |  |  |  |  |
| Total | 2979 | 2822 | 560 | 386 | 315 |
| Deamidable (%) | 75.16 | 72.82 | 61.25 | 65.54 | 65.71 |
| Deamidated (%) | 32.69 ^a^ | 50.02 ^b^ | 45.77 ^b^ | 67.59 ^c^ | 61.84 ^c^ |
|  |  |  |  |  |  |
| **Collagen Deamidation sites** |  |  |  |  |  |
| Total sequenced amino acids | 9442 | 6251 | 3491 | 2116 | 1994 |
| Deamidable amino acids (%) | 7.17 | 7.41 | 4.1 | 3.64 | 3.91 |
| N (%) | 45.2 | 44.49 | 36.36 | 41.56 | 41.03 |
| Q (%) | 54.8 | 55.51 | 63.64 | 58.44 | 58.97 |
| Deamidated sites (%) | 59.53 ^b^ | 67.6 ^a^ | 53.15 ^b^ | 80.52 ^a^ | 79.49 ^a^ |
| Deamidated N-sites (%) | 67.97 | 68.93 | 71.15 | 78.13 | 84.38 |
| Deamidated Q-sites (%) | 52.56 | 66.54 | 42.86 | 82.22 | 76.09 |
| ^a, b, c^ Values with different superscripts significantly differ between groups (*P* < 0.05) by Tukey’s WSD test. The values are presented alphabetically. | | | | | |

**Table S7:** The number of nucleus-like structures collected from mammoth tissues**.**

| Experiment | Detailed data represented in | Date of experiment | Weight of tissue | No. of nuclei found | Comment |
| --- | --- | --- | --- | --- | --- |
|  |  |  |  |  |  |
| 1st | Table S9 | 2015.9.10 | 28.5mg, 27.5mg | 11 |  |
| 2nd | Table S9 | 2015.9.11 | 24.9mg | 28 |  |
| 3rd | Table S9 | 2015.11.18 | 37.8mg, 62.1mg | 4 |  |
| 4th | Table S10 | 2016.1.7 | 29.0mg, 29.0mg | 23 | Nuclei were used for various assays including live-cell imaging and immunostaining. |
| 5th | Table S10 | 2016.1.8 | 34.7mg | 22 | Nuclei were used for various assays including live-cell imaging and immunostaining. |
| Total |  |  | 273.5 mg | 88 |  |

**Table S8: Summary of nuclear transfer, imaging and nuclear integrity.**

| Animal | Total | NT | | Imaging | | | | | | | |
| --- | --- | --- | --- | --- | --- | --- | --- | --- | --- | --- | --- |
|  |  | Dead | Survived | Imaged | Dead | Fragmented | Analysed | Before activation | | After activation | |
|  |  |  |  |  |  |  |  | H2B incorporation | Spindle formation | PN-like structure formation | |
|  |  |  |  |  |  |  |  |  |  | mouse | donor |
| Elephant | 40 | 4 | 36 | 28 (100) ^a^ | 0 (0) | 0 (0) | 28 (100) | 27 (96.4) | 26 (92.9) | 19 (67.9) | 11 (39.3) |
| Mammoth | 43 | 9 | 34 | 34 (100) | 5 (14.7) | 5 (14.7) | 24 (100) | 21 (87.5) | 5 (20.8) | 19 (79.2) | 1 (4.2) |

^a^ Remaining 8 embryos were used for another study.

**Table S9: Nuclear states of nuclear transfer oocytes reconstructed by transferring mammoth nuclei into mouse oocytes.**

| Experiment | Nucleus No. | Status after injection | Status during imaging | Before activation | | | After activation | |
| --- | --- | --- | --- | --- | --- | --- | --- | --- |
|  |  |  |  | H2B incorporation | Change in fluorescent intensity | Spindle reorganisation | Pronuclear  formation | |
|  |  |  |  |  |  |  | Mouse | Mammoth |
| 1st | #1 | dead | n.d. | n.d. | n.d. | n.d. | n.d. | n.d. |
|  | #2 | survived | dead | + | n.d. | n.d. | n.d. | n.d. |
|  | #3 | survived | survived | - | - | - | + | - |
|  | #4 | survived | survived | + | increase | - | + | - |
|  | #5 | survived | fragmented | + | constant | - | + | - |
|  | #6 | lost | n.d. | n.d. | n.d. | n.d. | n.d. | n.d. |
|  | #7 | survived | survived | + | increase | - | + | - |
|  | #8 | survived | dead | + | n.d. | n.d. | n.d. | n.d. |
|  | #9 | survived | fragmented | - | - | - | + | - |
|  | #10 | survived | dead | + | n.d. | n.d. | n.d. | n.d. |
|  | #11 | survived | survived | + | constant | + | + | - |
| 2nd | #12 | survived | fragmented | + | increase | - | + | - |
|  | #13 | survived | fragmented | + | increase | - | + | - |
|  | #14 | survived | survived | + | increase | - | + | - |
|  | #15 | survived | fragmented | + | increase | - | + | - |
|  | #16 | survived | survived | - | - | - | + | - |
|  | #17 | survived | survived | + | increase | - | - | - |
|  | #18 | survived | dead | + | n.d. | n.d. | n.d. | n.d. |
|  | #19 | dead | n.d. | n.d. | n.d. | n.d. | n.d. | n.d. |
|  | #20 | survived | survived | + | increase | - | - | - |
|  | #21 | survived | survived | - | - | - | + | - |
|  | #22 | survived | survived | + | constant | - | - | - |
|  | #23 | dead | n.d. | n.d. | n.d. | n.d. | n.d. | n.d. |
|  | #24 | dead | n.d. | n.d. | n.d. | n.d. | n.d. | n.d. |
|  | #25 | dead | n.d. | n.d. | n.d. | n.d. | n.d. | n.d. |
|  | #26 | survived | survived | + | constant | + | - | - |
|  | #27 | survived | survived | + | increase | - | - | - |
|  | #28 | survived | survived | + | increase | - | + | - |
|  | #29 | survived | survived | + | increase | - | - | - |
|  | #30 | survived | survived | + | constant | - | - | - |
|  | #31 | survived | survived | + | increase | - | - | - |
|  | #32 | dead | n.d. | n.d. | n.d. | n.d. | n.d. | n.d. |
|  | #33 | survived | survived | + | constant | - | - | - |
|  | #34 | survived | survived | + | constant | - | - | - |
|  | #35 | survived | out of view | + | decrease | - | - | - |
|  | #36 | dead | n.d. | n.d. | n.d. | n.d. | n.d. | n.d. |
|  | #37 | survived | survived | + | decrease | - | + | - |
|  | #38 | dead | n.d. | n.d. | n.d. | n.d. | n.d. | n.d. |
|  | #39 | survived | survived | + | constant | - | + | - |
| 3rd | #40 | survived | survived | + | constant | + | + | - |
|  | #41 | survived | survived | + | constant | + | + | + * |
|  | #42 | survived | survived | + | constant | + partially | + | - ** |
|  | #43 | survived | survived | + | decrease | - | + | - |
| * Small nucleus-like structures were formed at the timing of PN formation of mouse female chromosome.  ** A piece of chromosome entered into mouse PN. | | | | | | | | |

| **Table S10: Summary of DNA damage quantification in mammoth NLS by DDI method.** | | | | | | | |
| --- | --- | --- | --- | --- | --- | --- | --- |
| Exp. | Embryo No. | Status after injection | Status during imaging | Before activation | | After activation | |
|  |  |  |  | H2B incorporation | γH2A.X signal | Pronuclear formation | |
|  |  |  |  |  |  | Mouse | Mammoth |
| 1st | #1 | survived | survived | + | + | - | - |
|  | #2 | survived | survived | + | + | - | - |
|  | #3 | survived | fragmented | n.d. | n.d. | n.d. | n.d. |
|  | #4 | survived | survived | + | + | + | - |
|  | #5 | survived | fragmented | + | + | n.d. | n.d. |
|  | #6 | dead | n.d. | n.d. | n.d. | n.d. | n.d. |
|  | #7 | survived | survived | + | + | - | - |
|  | #8 | survived | survived | + | + | + | n.d. |
| 2nd | #9 | survived | survived | + | + | - | - |
|  | #10 | survived | survived | + | + | + | - |
|  | #11 | survived | survived | + | + | + | - |
|  | #12 | survived | survived | + | + | + | - |
|  | #13 | survived | survived | + | + | + | - |
|  | #14 | survived | survived | + | + | + | - |
|  | #15 | survived | survived | + | + | - | - |
|  | #16 | survived | survived | + | + | - | - |

**References:**

22. Robinson, N. E. & Robinson, A. B. Prediction of protein deamidation rates from primary and three-dimensional structure. *Proc. Natl. Acad. Sci. U. S. A.* **98**, 4367–4372 (2001).

23. Rasmussen, M. *et al*. Ancient human genome sequence of an extinct Palaeo-Eskimo. *Nature* **463**, 757-762 (2010).

24. Li, H. & Durbin, R. Fast and accurate short read alignment with Burrows-Wheeler transform. *Bioinformatics* **25**, 1754-1760 (2009).

25. Li, H. *et al*. 1000 Genome Project Data Processing Subgroup, The Sequence Alignment/Map format and SAMtools. *Bioinformatics* **25**, 2078-2079 (2009).

26. Langmead, B., Salzberg, S. L. Fast gapped-read alignment with Bowtie 2. *Nat. Methods* **9**, 357-359 (2012).

27. Jónsson, H., Ginolhac, A., Schubert, M., Johnson, P. L. & Orlando, L. mapDamage2.0: fast approximate Bayesian estimates of ancient DNA damage parameters. *Bioinformatics* **29**, 1682-1684 (2013).

28. Tamura, K., Stecher, G., Peterson, D., Filipski, A. & Kumar, S. MEGA6: Molecular Evolutionary Genetics Analysis version 6.0. *Mol. Biol. Evol.* **30**, 2725-2729 (2013).

29. Kimura, H., Hayashi-Takanaka, Y., Goto, Y., Takizawa, N. & Nozaki, N. The organization of histone H3 modifications as revealed by a panel of specific monoclonal antibodies. *Cell Struct. Funct.* **33**, 61-73 (2008).

30. Hayashi-Takanaka, Y. *et al*. Tracking epigenetic histone modifications in single cells using Fab-based live endogenous modification labeling. *Nucleic Acids Res.* **39**, 6475–6488 (2011).

31. Kishigami, S. & Wakayama, T. Efficient strontium-induced activation of mouse oocytes in standard culture media by chelating calcium. *J. Reprod. Dev.* 53, 1207-1215 (2007).

32. Yamagata, K. & Ueda, J. Long-term live-cell imaging of mammalian preimplantation development and derivation process of pluripotent stem cells from the embryos. *Dev. Growth Differ.* 55, 378-389 (2013).

Supplementary Table S2 (separate file): List of proteins identified in 1) the mammoth bone marrow and 2) the mammoth muscle.

Supplementary Table S3 (separate file): List of the identified proteins related to haematopoiesis in 1) the bone marrow and 2) the muscle.

Supplementary Table S4 (separate file): Compatible amino acid substitutions identified in the woolly mammoth by genomic and proteomic analyses.

Supplementary Table S5 (separate file): Summary of comprehensive analysis of post-translationally modified peptides.

Movie S1: Time-lapse image of histone H2B-mCherry (H2B, red) and EGFP-EB1 (EB1, green) dynamics in oocytes immediately after the injection of elephant (A) and mammoth (B and C) nuclei. Time from the starting point of imaging is represented as (h:min). Note that H2B incorporation and spindle formation in the elephant and mammoth nuclei were seen along with the stable appearance of metaphase II plate (A and C). H2B incorporation to the mammoth nucleus was seen, but spindle formation was not observed (B).

Movie S2: Time-lapse image of nuclear formation processes after the activation of NT oocytes reconstructed with an elephant or mammoth somatic nucleus. Images are shown by histone H2B-mCherry fluorescence. Small pronucleus-like structures were budded from a part of both elephant and mammoth nuclei injected. Time from the starting point of imaging is represented as (hr:min).

Movie S3: Time-lapse image of chromosome (H2B, red) and spindle (EB1, green) dynamics in an oocyte immediately after the injection of mammoth nucleus. A part of chromosome was detached from the injected mammoth nucleus. Time from the starting point of imaging is represented as (hr:min).

Movie S4: Time-lapse image of mammoth chromosome (H2B, red) and spindle (EB1, green) dynamics immediately after the activation of the same oocyte with the Supplementary Movie 5. Interestingly, a mammoth chromosome piece was captured by mouse anaphase II spindle at around 4:00 and it entered into mouse pronucleus at around 5:00. Time from the starting point of imaging is represented as (hr:min).

Movie S5: Kinetics of DSBs in a mammoth nucleus immediately after injection into mouse oocyte. Fluorescently labelled Fab fragment against phosphorylated histone H2A.X (pH2A.X, green) and H2B-mCherry mRNA (H2B) were used for the visualisation and quantification of the break. The mammoth nucleus with the highest (strong) and lowest (weak) broken genome examined are shown. Time from the starting point of imaging is represented as (hr:min).

Movie S6: Kinetics of DSBs in the mammoth nucleus after oocyte activation. Fluorescently labelled Fab fragment against phosphorylated histone H2A.X (pH2A.X, green) and H2B-mCherry mRNA (H2B) were used for the visualisation and quantification of the break. Mammoth nuclei in which the level of DSBs were increased (Strong) and decreased (Weak) during pronuclear formation are shown. Time from the starting point of imaging is represented as (hr:min).
